# High-Throughput Determination of 210 Pesticide Residues in Gherkins by QuEChERS Coupled with LC-MS/MS and GC-MS/MS

**DOI:** 10.3390/molecules31081248

**Published:** 2026-04-09

**Authors:** Mehmet Keklik, Eylem Odabas, Tuba Buyuksirit-Bedir, Ozgur Golge, Miguel Ángel González-Curbelo, Bulent Kabak

**Affiliations:** 1 Food Control Laboratory, Yonca Gıda, Şehzadeler, Manisa 45030, Türkiye; mkeklik@yildiz.edu.tr; 2 Department of Food Engineering, Faculty of Engineering and Natural Sciences, Hitit University, Corum 19030, Türkiye; eylem.odabas@ogrenci.hitit.edu.tr (E.O.); tubabuyuksirit@hitit.edu.tr (T.B.-B.); 3 Department of Gastronomy and Culinary Arts, Faculty of Tourism, Alanya Alaaddin Keykubat University, Alanya 07425, Türkiye; ozgur.golge@alanya.edu.tr; 4 Departamento de Ciencias Básicas, Facultad de Ingeniería, Universidad EAN, Calle 79 n11-45, Bogotá 110221, Colombia

**Keywords:** multi-residue analysis, multi-class pesticides, food contaminants, chromatographic techniques, trace-level determination, method performance evaluation

## Abstract

Pesticide residues represent an important group of chemical contaminants in agricultural commodities and require reliable analytical strategies for accurate monitoring. In this study, a high-throughput analytical workflow was applied for the determination of 210 pesticide residues in gherkins. Sample preparation was performed using the quick, easy, cheap, effective, rugged, and safe (QuEChERS) method, including extraction followed by dispersive solid-phase extraction clean-up. Residue determination was carried out using liquid chromatography–tandem mass spectrometry (LC-MS/MS) and gas chromatography–tandem mass spectrometry (GC-MS/MS). The analytical methods were comprehensively validated in the gherkin matrix in accordance with the SANTE 11312/2021 v2 guidelines. Limits of quantification were ≤0.01 mg kg^−1^ for all compounds. Recovery values ranged from 75.7% to 113.7%, while precision values remained below 20%, demonstrating satisfactory method accuracy and precision. Expanded measurement uncertainty values ranged between 7.6% and 41.3%, confirming the robustness of the validated analytical workflow. The validated methods were subsequently applied to a large-scale monitoring dataset comprising 905 gherkin samples collected from five major production regions in Türkiye. Pesticide residues were detected in 67.6% of the analysed samples, and 37 different compounds were identified. The most frequently detected pesticides were flonicamid (36.2%) and propamocarb (27.5%). Multi-residue contamination was frequently observed, reflecting complex pesticide application patterns in gherkin cultivation systems. Although chronic exposure estimates remained well below toxicological thresholds for both adults and children, certain exposure scenarios indicated that acute exposure for children may warrant further attention.

## 1. Introduction

Gherkins (*Cucumis sativus* L.), belonging to the Cucurbitaceae family, represent an economically important horticultural crop widely used for both fresh consumption and industrial processing, particularly for pickling and brining. Although no formal taxonomic distinction exists between table cucumbers and gherkins, these products are commonly differentiated based on fruit morphology. Gherkins are typically harvested at a fruit length of 5–10 cm, whereas table cucumbers are generally harvested at larger and more variable sizes (10–50 cm), depending on cultivar and intended use [1].

Throughout the growing season, gherkin crops are exposed to a wide spectrum of biotic stressors, including fungal diseases and insect infestations, which can significantly affect crop productivity and quality. To limit crop losses associated with these pressures, intensive applications of insecticides and fungicides have been widely implemented in gherkin production systems [2]. However, pesticide residues are not confined to target organisms and may disperse into non-target crops and environmental compartments [3]. The presence of these residues has raised persistent concerns regarding food safety and ecosystem integrity [4,5]. Epidemiological evidence has indicated associations between pesticide exposure and oxidative stress, as well as increased risks of neurodegenerative disorders, endocrine disruption, and several types of cancer [6,7].

In response to these health concerns, international regulatory authorities, including the Food and Agriculture Organization of the United Nations (FAO), the World Health Organization (WHO), and the European Food Safety Authority (EFSA), have established legal limits and toxicological reference values for pesticide residues in food commodities [8,9]. Maximum residue levels (MRLs), acceptable daily intake (ADI), and acute reference dose (ARfD) values constitute the key parameters in regulatory frameworks used to evaluate pesticide residues and their potential dietary risks. According to the EFSA monitoring report for 2023, 96.3% of food products marketed within the European Union (EU) complied with established MRLs, while exceedances were detected in 3.7% of samples [9].

The accurate determination of pesticide residues in agricultural products requires sensitive and reliable analytical methods. In particular, efficient extraction from complex food matrices represents a critical step in multi-residue pesticide analysis. The quick, easy, cheap, effective, rugged, and safe (QuEChERS) method has emerged as one of the most effective extraction strategies due to its operational simplicity, rapid processing time, and high extraction efficiency [10]. Its compatibility with both liquid chromatography (LC) and gas chromatography (GC) methods, combined with low solvent consumption and consistently high recovery rates, has established QuEChERS as a standard technique in contemporary pesticide residue analysis [11]. Compared with conventional extraction procedures such as liquid–liquid extraction and solid-phase extraction, QuEChERS offers a markedly simplified workflow and reduced analysis time, which has facilitated its extensive use in multi-residue monitoring studies [10,12].

Following extraction, pesticide residues are typically quantified using chromatographic techniques coupled with mass spectrometry. Gas chromatography–tandem mass spectrometry (GC-MS/MS) provides effective separation and detection for volatile and thermally stable pesticide compounds, whereas liquid chromatography–tandem mass spectrometry (LC-MS/MS) exhibits superior analytical performance for polar and thermally labile pesticides [10,13]. These tandem mass spectrometric systems equipped with triple quadrupole mass analyzers enable highly selective and sensitive quantification of numerous pesticide residues simultaneously, even at trace concentration levels in complex food matrices [13]. Thus, chromatographic techniques coupled with tandem mass spectrometry provide substantial analytical advantages over rapid screening methods, particularly in the reliable quantification of low-level pesticide residues [11].

Although gherkins are widely consumed and play a significant role in the food processing industry, comprehensive monitoring data on pesticide residues in this commodity remain limited. Most studies have focused on table cucumbers, assessing pesticide occurrence and associated health risks [14,15,16,17,18,19], while investigations specifically targeting gherkins are scarce [2]. This disparity constrains reliable characterization of dietary exposure and results in uncertainty in food safety evaluations for this commodity.

The present study was conducted to address critical knowledge gaps in pesticide residue monitoring in gherkins produced across Türkiye by implementing a high-throughput QuEChERS-based extraction procedure combined with LC-MS/MS and GC-MS/MS analysis. Furthermore, systematic sampling was conducted across five major production provinces, Afyonkarahisar, Balıkesir, İzmir, Manisa, and Mardin, addressing regional data gaps and enabling the generation of region-specific risk profiles, which were previously unavailable in the literature. In addition to providing a comprehensive dataset covering 210 pesticide residues (LC-amenable and GC-amenable), the study integrated quality-based evaluation using the Index of Quality for Residues (IqR) and multiple risk assessment frameworks, including deterministic and probabilistic approaches based on Monte Carlo simulation (MCS), as well as risk ranking score evaluations, with both chronic and acute dietary risks assessed separately for adults and children, thereby offering a multifaceted and robust assessment of potential consumer health risks. The validated analytical procedures employed, the integration of complementary risk assessment approaches, and the extensive regional coverage distinguish the present study from earlier reports, highlighting its unique contributions to characterising both the occurrence of pesticide residues and their potential health implications in gherkins.

## 2. Results and Discussion

### 2.1. Method Validation Data

Method validation ensures that the analytical procedures are reliable and fit-for-purpose, providing a robust basis for subsequent pesticide residue detection. Method selectivity was confirmed by the absence of interfering peaks within a ±2.5% retention time window around the expected retention times of the target analytes. Retention times of the analytes deviated by less than 0.1 min from the matrix-matched calibration standards, and relative ion ratios were maintained below 30%, in accordance with the SANTE 11312/2021 v2 guidelines [20]. Across the entire concentration range from 0.005 to 0.25 mg kg^−1^, all target analytes exhibited highly linear responses with coefficients of determination (*R*^2^) greater than 0.999. The use of matrix-matched calibration standards compensated for potential matrix effects arising from the complex gherkin matrix.

**Figure 1:**
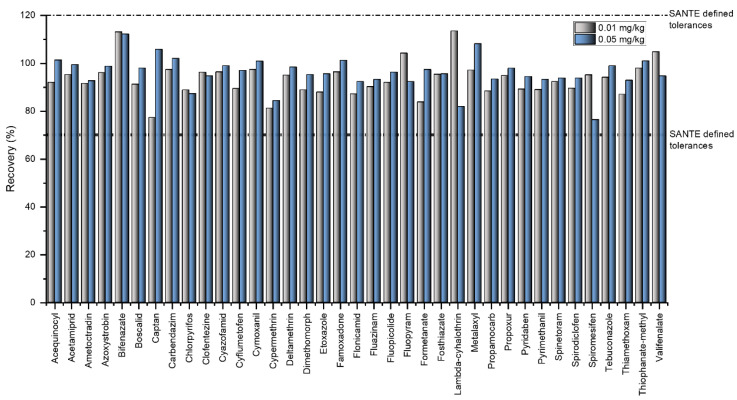
Recovery values (%) of 37 pesticide residues in spiked gherkin samples at two fortification levels obtained during method validation. Dashed lines indicate the acceptable recovery range (70–120%) specified in the SANTE 11312/2021 v2 guidelines.

**Figure 2:**
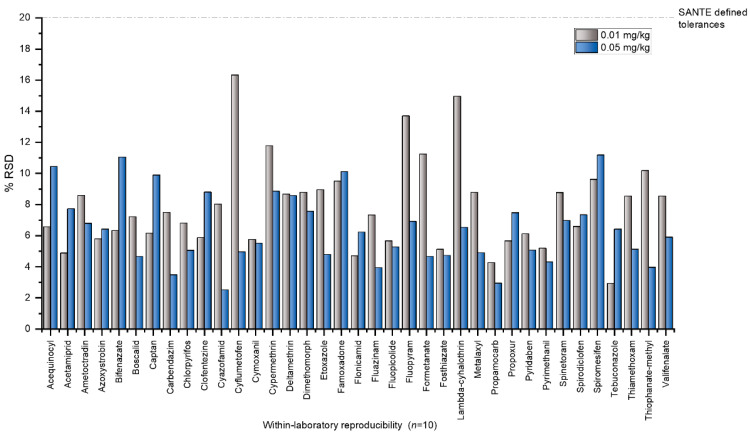

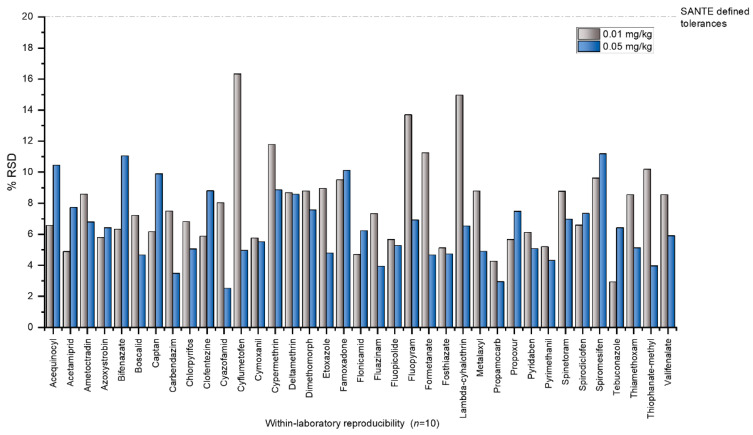
The RSDr and RSD_WR_ values for pesticides in spiked gherkin samples at two fortification levels. The dashed line indicates the maximum acceptable precision (20%) according to the SANTE 11312/2021 v2 guidelines.

The method validation results for recoveries and precision are summarised in Table S1. In terms of sensitivity, all analytes had a limit of quantification (LOQ) ≤ 0.01 mg kg^−1^ by both LC-MS/MS and GC-MS/MS, indicating that the method is fit-for-purpose for pesticide residue analyses. Recovery experiments confirmed satisfactory trueness for both LC-MS/MS and GC-MS/MS methods. At the lower fortification level (0.01 mg kg^−1^), recoveries for LC-MS/MS ranged from 79.8% to 113.7%, whereas at the higher level (0.05 mg kg^−1^) they ranged from 76.4% to 112.2%. For GC-MS/MS, recoveries varied between 77.3% and 110.8% at 0.01 mg kg^−1^, and between 75.7% and 112.7% at 0.05 mg kg^−1^. Under repeatability conditions (within-day, n = 5), relative standard deviation of repeatability (RSDr) values for LC-MS/MS ranged from 2.79% to 16.20% at the lower fortification level (0.01 mg kg^−1^) and from 1.78% to 11.22% at the higher fortification level (0.05 mg kg^−1^). For GC-MS/MS, RSDr values varied between 3.54% and 8.43% at 0.01 mg kg^−1^, and between 2.61% and 11.22% at 0.05 mg kg^−1^. Within-laboratory reproducibility (RSD_WR_), assessed over five days by two independent analysts, ranged from 1.78% to 15.6% for LC-MS/MS at 0.01 mg kg^−1^ and from 2.10% to 17.66% at 0.05 mg kg^−1^. The RSD_WR_ values for GC-MS/MS were 2.90–17.2% at the lower fortification level and 3.12–19.76% at the higher level. These results are fully consistent with the performance criteria defined in the SANTE 11312/2021 v2 guidelines [20], which specify acceptable recoveries of 70–120% and RSDr/RSD_WR_ ≤ 20% for pesticide residue analyses. The distribution of recoveries and precision parameters for the 37 pesticides detected in gherkin samples is illustrated in Figure 1 and Figure 2, respectively. Measurement uncertainty, representing the expected range of the true value with a given confidence level, was assessed for all analytes, yielding expanded uncertainties (*k* = 2) between 7.6% and 41.3%, which underscores the precision and reliability of the validated analytical procedures [20].

### 2.2. Distribution of Pesticide Residues in Gherkins

**Figure 3:**
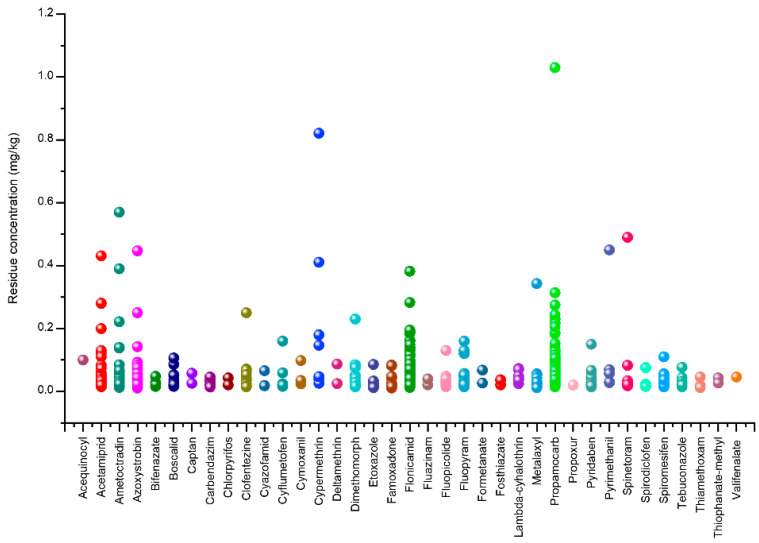
Distribution of pesticide residues in gherkin samples determined by LC-MS/MS and GC-MS/MS.

**Figure 4:**
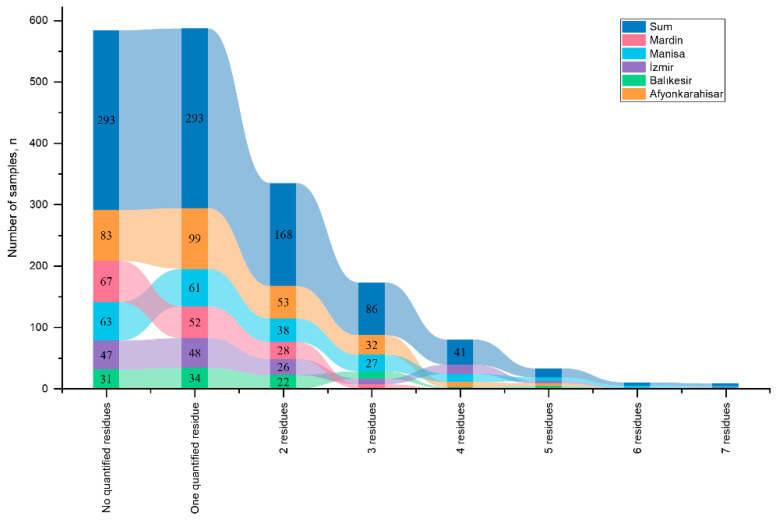
Distribution of single and multiple pesticide residues in gherkin samples from five production regions of Türkiye.

Building on the validated analytical results, the prevalence and concentration of pesticide residues across provinces are presented, providing the necessary data for subsequent quality assessment and risk characterization. A total of 905 gherkin samples collected in 2025 from five major production provinces in Türkiye, Afyonkarahisar (n = 280), Balıkesir (n = 104), İzmir (n = 152), Manisa (n = 214), and Mardin (n = 155), were analysed for the presence of 210 pesticide residues using LC-MS/MS and GC-MS/MS. The distribution patterns of the detected residues and their corresponding concentration ranges are presented in Figure 3. Measurable pesticide residues were detected in 612 samples (67.6%), whereas no detectable residues were observed in the remaining 293 samples. Among the contaminated samples, 73 exceeded the EU MRLs. Multiple residues were detected in a considerable proportion of the samples: 319 samples contained more than one pesticide, whereas 293 samples contained a single residue. Among the multi-residue samples, two residues were detected in 168 samples, three residues in 86 samples, four residues in 41 samples, five residues in 15 samples, six residues in five samples, and seven residues in four samples (Figure 4). These findings indicate that the co-occurrence of pesticide residues is a common feature in the analysed gherkin samples.

A total of 37 different pesticide residues were identified in the analysed samples, including 11 unauthorised active substances (carbendazim, chlorpyrifos, clofentezine, dimethomorph, famoxadone, propoxur, spinetoram, spirodiclofen, spiromesifen, thiamethoxam, and thiophanate-methyl). Among the detected compounds, 18 were classified as fungicides, 17 as insecticides, and two as acaricides. The diversity of detected residues varied considerably among the production regions. The highest diversity was observed in samples collected from Manisa (n = 26), followed by İzmir (n = 22), Afyonkarahisar (n = 20), Mardin (n = 16), and Balıkesir (n = 14). Unauthorised pesticide usage was most frequently observed in Afyonkarahisar and Manisa (n = 7 in each province) regions, followed by İzmir and Mardin (n = 5 each) and Balıkesir (n = 3).

**Figure 5:**
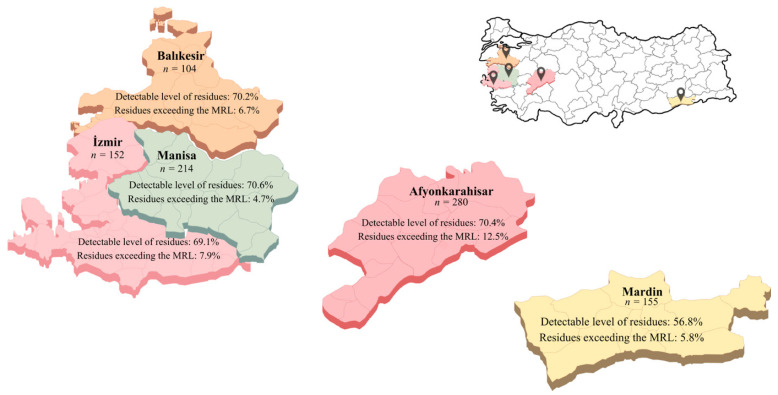
Detection frequencies and EU MRL exceedances of pesticide residues in gherkin samples across five production regions.

The prevalence of detectable pesticide residues also varied among the provinces. Residues were detected in 70.6% of the samples from Manisa, 70.4% from Afyonkarahisar, 70.2% from Balıkesir, 69.1% from İzmir, and 56.8% from Mardin. A chi-square test confirmed that these differences were statistically significant (χ^2^ = 10.61, *p* = 0.031), primarily driven by the lower detection frequency observed in Mardin relative to the other provinces. Despite these regional differences, the overall detection frequency exceeded 50% in all provinces, indicating widespread pesticide application in gherkin cultivation. Similarly, the proportion of samples exceeding EU MRLs differed significantly among provinces (χ^2^ = 11.12, *p* = 0.025), with the highest number of non-compliant samples was observed in Afyonkarahisar (n = 35), followed by İzmir (n = 12), Manisa (n = 10), Mardin (n = 9), and Balıkesir (n = 7) (Figure 5). These findings suggest that both the intensity of pesticide usage and the degree of compliance with recommended agricultural practices may vary substantially across production regions.

The most frequently detected pesticide was flonicamid, occurring in 36.2% of the analysed samples. It was the most prevalent residue in samples from Manisa (52.8%) and Balıkesir (39.4%), whereas it ranked second most frequently detected in İzmir (32.2%), Afyonkarahisar (30.0%), and Mardin (26.5%). Flonicamid concentrations ranged from 0.013 to 0.382 mg kg^−1^, with a mean concentration of 0.048 mg kg^−1^. The high detection frequency of flonicamid may be associated with its widespread use for the control of sap-sucking pests in cucurbit crops, particularly aphids and whiteflies [21].

Propamocarb ranked second in terms of detection frequency, occurring in 27.5% of the analysed samples. It was the most prevalent residue in samples collected from Afyonkarahisar (41.8%) and İzmir (37.5%), while it ranked second in Manisa (29.9%), fourth in Mardin (3.9%), and seventh in Balıkesir (4.8%). The levels of propamocarb in gherkins ranged from 0.015 to 1.030 mg kg^−1^, with a mean concentration of 0.056 mg kg^−1^. The frequent occurrence of propamocarb likely reflects its extensive application for the management of downy mildew in cucurbit production systems [22].

Acetamiprid was the third most prevalent residue, being present in 13.3% of the analysed samples. The concentrations of acetamiprid ranged between 0.015 and 0.331 mg kg^−1^, with a mean concentration of 0.040 mg kg^−1^. The occurrence of acetamiprid may be attributed to its widespread application for the control of sap-sucking pests, particularly aphids, in cucurbit production systems [2].

Azoxystrobin was identified as the fourth most frequently detected residue, occurring in 11.2% of the samples at concentrations ranging from 0.011 to 0.447 mg kg^−1^ (mean = 0.038 mg kg^−1^). The presence of azoxystrobin likely reflects its extensive use for the management of fungal diseases, such as powdery and downy mildews, in cucurbit crops [23].

In addition to these frequently detected residues, several other pesticides were detected at lower frequencies (≤10%). These compounds included ametoctradin (8.8%, 0.013–0.570 mg kg^−1^), fluopyram (3.7%, 0.015–0.160 mg kg^−1^), spiromesifen (3.7%, 0.015–0.110 mg kg^−1^), dimethomorph (3.4%, 0.015–0.230 mg kg^−1^), metalaxyl (2.9%, 0.012–0.343 mg kg^−1^), fluopicolide (2.5%, 0.015–0.130 mg kg^−1^), pyridaben (2.3%, 0.015–0.150 mg kg^−1^), famoxadone (2.1%, 0.011–0.084 mg kg^−1^), clofentezine (1.6%, 0.015–0.250 mg kg^−1^), boscalid (1.4%, 0.016–0.106 mg kg^−1^), and tebuconazole (1.0%, 0.015–0.077 mg kg^−1^), together with several additional compounds detected at frequencies below 1% (Table S2). Although these compounds were detected less frequently, their presence further highlights the complex mixture of pesticide residues occurring in gherkin production systems.

A review of the available literature indicates that relatively few studies have specifically investigated pesticide residues in gherkin samples, whereas most studies have focused on cucumbers. When the present findings were compared with the limited number of previously reported studies on gherkins, a notably higher diversity and frequency of pesticide residues were observed in the analysed samples. For example, Golge et al. [2] analysed 203 gherkin samples collected in Türkiye between 2017 and 2019 for 109 pesticide residues and reported measurable residues in 42.4% of the samples, with only 15 different pesticides detected. In contrast, the present study identified 37 distinct residues in 67.6% of the analysed samples, indicating a substantially broader spectrum of pesticide occurrence. Moreover, multi-residue contamination was considerably more common in the present dataset, suggesting increasingly complex pesticide application patterns in gherkin production. Another notable difference concerns the identity of the most frequently detected pesticide residues. In the earlier study, chlorothalonil was reported as the dominant compound (32%), whereas it was not detected in the present study. This difference may be partly explained by regulatory changes affecting pesticide authorisations. Chlorothalonil, a widely used fungicide in vegetable production, lost its approval in the EU in 2019 due to concerns regarding potential environmental and health risks [24]. Following its withdrawal, the patterns of fungicide usage in gherkin cultivation have shifted. In the present study, propamocarb was the most frequently detected fungicide (27.2%), reflecting the increased use of alternative compounds in pest management strategies.

Studies conducted on cucumbers in Türkiye and other countries have also reported varying levels of pesticide contamination. In a previous study in Türkiye, pesticide residues were detected in 13.5% of 400 cucumber samples, with propamocarb, acetamiprid, and dimethomorph being the most frequently identified compounds [25]. International investigations have similarly reported considerable variability in residue occurrence depending on geographical region and production system. For example, diazinon, acetamiprid, and imidacloprid were frequently detected in cucumber samples from Iran, although most concentrations remained below established MRL values [14,17,18]. In Egypt, Bangladesh, and Kazakhstan, pesticide residues were reported in 10–100% of analysed samples, with occasional exceedances of regulatory limits [16,26,27]. Similarly, a large-scale investigation in China demonstrated higher pesticide occurrence in greenhouse cucumbers (14 different residues) compared with open-field production systems (10 different residues) [19]. A recent meta-analysis further confirmed that acetamiprid, diazinon, and chlorpyrifos are among the most frequently detected pesticide residues in cucumbers worldwide, although considerable country-specific variations were observed [28]. Information from the Rapid Alert System for Food and Feed (RASFF) also indicates three notifications concerning gherkin products, involving chlorpyrifos (two cases) and metalaxyl (one case) [29].

When compared with these previously reported national and international studies, the present dataset reveals a markedly higher diversity of and detection frequency for pesticide residues in gherkins. Measurable residues were detected in more than two-thirds of the analysed samples, and multi-residue contamination was widely observed, indicating increasingly complex pesticide application patterns in gherkin production systems. Furthermore, the detection of unauthorised active substances and the occurrence of samples exceeding EU MRL values highlight potential food safety concerns and suggest that compliance with recommended agricultural practices may vary among production regions. A further notable observation is the dominance of flonicamid and propamocarb among the detected residues. In contrast to earlier studies in which other compounds were reported as the most prevalent residues, the prominence of these pesticides may reflect recent shifts in pest management strategies and pesticide use patterns following regulatory changes that affected certain previously used compounds.

### 2.3. Quality Assessment of Gherkins

By applying the IqR to the previously reported residue concentrations, the impact of single and multiple residues on product quality can be quantitatively assessed. The quality and safety of agricultural commodities with respect to pesticide contamination are commonly evaluated based on compliance with MRLs. While MRL compliance reflects the legal acceptability of individual pesticide residues, the simultaneous occurrence of multiple residues within a single sample may influence overall product quality due to potential additive or synergistic effects. In this context, the IqR has been proposed as a valuable indicator of food quality, enabling the identification of low-quality products even when individual residues remain below their respective MRL [30]. By integrating the cumulative contribution of multiple residues detected in a sample, the IqR approach provides a more comprehensive assessment of pesticide contamination and its potential implications for food quality.

In the present study, the overall quality of the analysed gherkin samples was generally satisfactory according to the IqR classification criteria. Specifically, 32.4% of the samples were categorised as excellent quality (IqR = 0), 56.7% as good quality (IqR = 0–0.6), and 1.8% as adequate quality (IqR = 0.6–1), whereas the remaining 9.2% were classified as inadequate (IqR > 1). These findings indicate that the majority of the analysed samples fell within the acceptable quality categories, suggesting that, in most cases, pesticide residue levels remained within acceptable quality categories according to the IqR classification. Comparable assessments have been reported in studies conducted on cucumbers. For instance, Dong et al. [19] reported that approximately 35.6% of open-field cucumbers were classified as excellent quality (IqR = 0) and 64.4% as good quality (IqR = 0–0.6). In greenhouse-grown cucumbers, around 15% of samples were categorised as excellent, 74.4% as good, 4.4% as adequate (IqR = 0.6–1), and 6.1% as inadequate (IqR > 1). Although the present investigation focused on gherkin samples, the observed distribution of quality categories is broadly consistent with previously reported results for cucumbers, suggesting similar patterns of pesticide contamination across cucurbit production systems.

**Figure 6:**
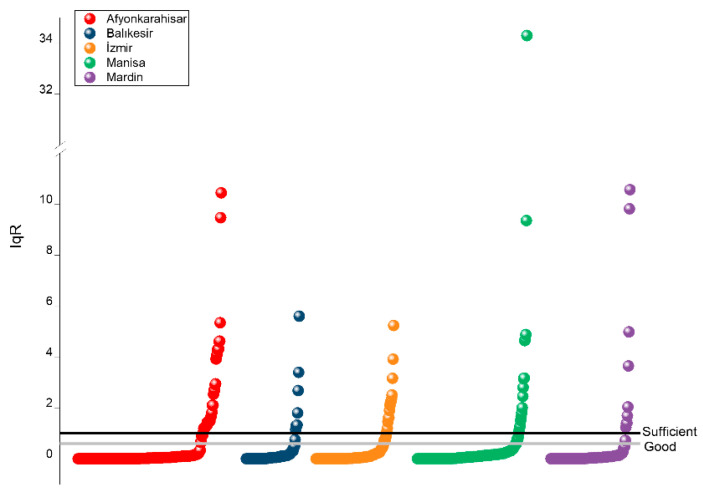
Quality classification of gherkin samples from different production regions based on the IqR.

Regional differences in quality categories were also observed among the analysed samples. Gherkins collected from Mardin exhibited the highest proportion of acceptable-quality samples (excellent or good), accounting for 93.5% of the analysed samples. Similarly high proportions were recorded in Balıkesir (91.3%) and Manisa (90.2%), followed by İzmir (86.8%) and Afyonkarahisar (84.3%) (Figure 6). Despite these regional variations, the majority of samples from all production provinces were categorised within the acceptable quality classes, indicating generally favorable pesticide residue profiles across the studied production areas.

A total of 83 samples were categorised as inadequate quality (IqR > 1). Among these samples, IqR values ranged between 1 and 2 in 39 samples, between 2 and 5 in 35 samples, between 5 and 10 in six samples, and exceeded 10 in three samples. The highest IqR value (34.3) was observed in a gherkin sample collected from Manisa. In this sample, metalaxyl was detected at a concentration substantially exceeding the corresponding MRL (0.343 mg kg^−1^), which directly contributed to the high IqR value. This observation illustrates how substantial exceedances of regulatory limits by individual pesticide residues can markedly increase the cumulative residue index and consequently influence the overall quality classification of the product.

### 2.4. Risk Ranking of Pesticide Residues

**Figure 7:**
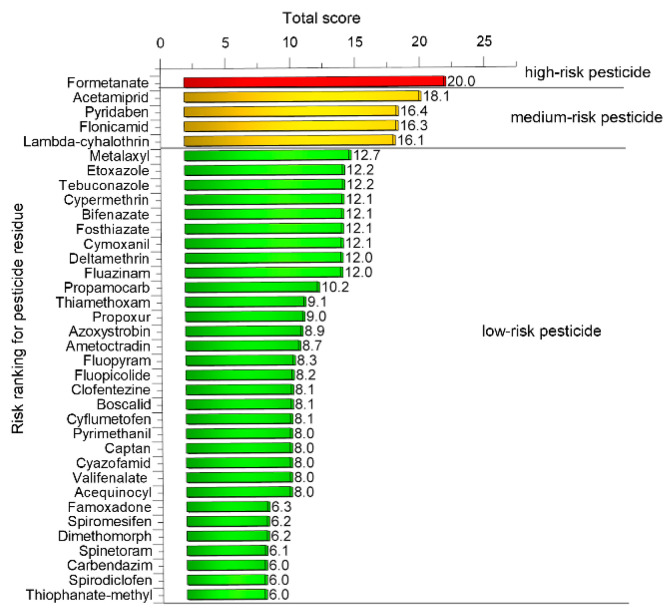
Risk ranking of pesticide residues detected in gherkin samples using a composite risk score. Pesticides were classified into high, medium, and low risk categories based on their total scores.

The risk ranking of individual pesticides was subsequently conducted using the measured residue concentrations and a matrix-based scoring system, enabling the identification of high-priority compounds for subsequent dietary exposure assessment. The risk ranking of pesticide residues detected in gherkin samples is shown in Figure 7. A total of 37 pesticide residues were evaluated, and their overall risk scores were categorised into three levels: high risk (score ≥ 20), medium risk (score 15–20), and low risk (score ≤ 15). However, chlorpyrifos was not included in the risk scoring due to the absence of an established ADI value. High-risk scores were observed only for formetanate, which received a score of 20. Medium-risk scores were assigned to four pesticides: acetamiprid (18.1), pyridaben (16.4), flonicamid (16.3), and lambda-cyhalothrin (16.1). Notably, all five pesticides receiving high- or medium-risk scores were insecticides, highlighting the predominance of compounds targeting insect pests among the highest-risk residues. The remaining 31 pesticides exhibited overall risk scores ranging from 6.0 to 12.7 and were thus classified as low risk. The distribution of these compounds indicates that, although a limited number of residues present moderate or high risk, the majority of detected compounds are associated with a relatively low potential for adverse effects. The predominance of low-risk scores highlights that, under the conditions studied, gherkin samples generally contain residues posing limited exposure concerns.

### 2.5. Deterministic Dietary Exposure and Risk Characterisation

**Figure 8:**
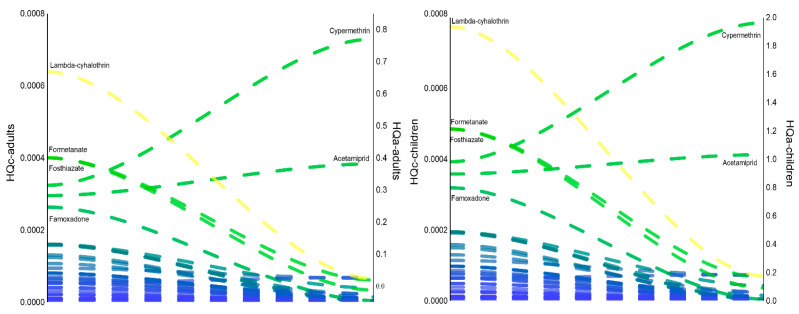
Deterministic assessment of dietary risk from pesticide residues in gherkin samples for adults and children, expressed as chronic and acute *HQs*.

Using the measured residue concentrations, cumulative chronic and acute exposure and hazard indices were calculated, thereby providing a quantitative hazard characterization of potential health risks. Chronic dietary exposure to individual pesticide residues via gherkin consumption was estimated for adults and children under both lower-bound (LB) and upper-bound (UB) scenarios (Table S3). Cumulative exposure to all detected pesticide residues was calculated as 9.8 × 10^−7^ mg kg^−1^ bw day^−1^ (LB) and 5.8 × 10^−5^ mg kg^−1^ bw day^−1^ (UB) for adults, and 1.6 × 10^−6^ mg kg^−1^ bw day^−1^ (LB) and 1.0 × 10^−4^ mg kg^−1^ bw day^−1^ (UB) for children. The chronic hazard quotient (*HQc*) of individual pesticides ranged from 5.9 × 10^−9^ to 1.7 × 10^−5^ (LB) and 1.5 × 10^−7^ to 6.4 × 10^−4^ (UB) for adults, and from 7.2 × 10^−9^ to 2.1 × 10^−5^ (LB) and 1.8 × 10^−7^ to 7.7 × 10^−4^ (UB) for children (Figure 8). Under the worst-case scenario, the chronic hazard index (*HIc*) was 0.0042 for adults and 0.0051 for children, indicating that chronic exposure via gherkin consumption is unlikely to pose a significant health risk to either population group. Among the detected pesticides, lambda-cyhalothrin (15%), formetanate (9%), and fosthiazate (9%) contributed most to the *HIc* in both age groups.

The acute hazard quotient (*HQa*) was assessed for the highest recorded concentrations of individual pesticides. For ten compounds lacking ARfD values (ametoctradin, azoxystrobin, boscalid, clofentezine, cyazofamid, etoxazole, propoxur, pyrimethanil, spirodiclofen, and valifenalate), ADI values were used for acute risk estimation (Table S3). *HQa* values ranged from 0.0003 to 0.8197 for adults and from 0.0007 to 1.9606 for children (Figure 8). The highest acute risk contributors were cypermethrin (*HQa*_adults_ = 0.8197; *HQa*_children_ = 1.9606) and acetamiprid (*HQa*_adults_ = 0.4303; *HQa*_children_ = 1.0292), with a single-child exposure scenario exceeding the threshold of concern. These results indicate that, while acute exposure via gherkin consumption does not constitute a significant risk for adult consumers, potential acute risk for children may warrant further attention.

### 2.6. Probabilistic Dietary Exposure and Risk Characterisation

To complement the deterministic assessment, a probabilistic evaluation was conducted using the MCS approach to provide a more comprehensive characterisation of variability and uncertainty in chronic dietary exposure. The distributions of *HIc* values for gherkins, together with the results of the sensitivity analysis (SA), are presented in Figure S1. Modeled exposure distributions indicated that *HIc* values associated with chronic pesticide intake via gherkin consumption exhibited comparable distributional characteristics for both adults and children. For adults, the *HIc* distribution approximated normality, with mean and median values converging at 0.0043. In children, a similar near-normal distribution was observed, with mean and median values of 0.0052. The close alignment of mean and median in both populations suggests the absence of substantial skewness and indicates that results were symmetrically distributed around the central tendency. The 5th and 95th percentiles for adults were calculated as 0.0041 and 0.0045, respectively, whereas corresponding percentiles for children were 0.0049 and 0.0055. Despite slightly higher *HIc* values in children relative to adults, even upper-percentile estimates remained well below the threshold of concern (*HIc* = 1), confirming that chronic dietary exposure through gherkin consumption is unlikely to pose long-term health risks in either age group.

SA further revealed that the uncertainty in *HIc* was predominantly driven by a limited subset of pesticides in both age groups. For adults, acetamiprid emerged as the primary contributor, accounting for approximately 42.5% of the total variance, followed by cypermethrin with a contribution of 38.1%. The remaining pesticides individually contributed only marginally to the overall variance. A similar pattern was observed in children, where acetamiprid accounted for approximately 41.5% of the total variance and cypermethrin for 38.4%, while all other pesticides exerted comparatively minor influences.

These findings highlight that the pesticides driving *HIc* are largely the same as those driving uncertainty, and they underscore how deterministic and probabilistic approaches can capture complementary aspects of risk. Overall, probabilistic analysis confirmed that cumulative chronic risk from gherkin consumption is predominantly shaped by acetamiprid and cypermethrin, whereas other pesticides contribute minimally to *HIc* variability.

Differences between the MCS results and the deterministic approach with respect to the types and relative contributions of pesticides to *HIc* can be attributed to the fundamental methodological differences between the two assessment frameworks. While the deterministic approach relies on mean input values representing point estimates, the MCS incorporates the variability and uncertainty associated with input parameters, thereby enabling the identification and quantification of the relative contributions of individual inputs to the overall variance of the risk output.

## 3. Materials and Methods

### 3.1. Chemicals and Reagents

High-performance liquid chromatography (HPLC)-grade acetonitrile and methanol were obtained from Sigma-Aldrich (Merck KGaA, Darmstadt, Germany). Analytical-grade glacial acetic acid (CH_3_COOH, ≥99.8%), ammonium formate (≥99%), formic acid (98–100%), and ultrapure water (LiChrosolv^®^) were supplied by Supelco (Merck KGaA, Darmstadt, Germany). QuEChERS sample preparation kits, compliant with AOAC Official Method 2007.01 [31], were purchased from CHROMAtific UG (Heidenrod, Germany).

Individual analytical pesticide reference standards (purity > 95%) were provided by Dr. Ehrenstorfer™ (Augsburg, Germany) and CPA Chem (Bogomilova, Bulgaria). Stock solutions comprising 210 target pesticides were prepared after evaluating the physicochemical properties of each analyte, including polarity, ionic character, solubility, and stability. Based on these properties, solvent systems were selected on an analyte-specific basis to ensure complete dissolution and adequate stability of the standards. Depending on the properties of each compound, individual stock standards were prepared in methanol, acetonitrile, acetone, ethyl acetate, or toluene. These stock solutions were subsequently used for the preparation of matrix-matched calibration standards and for spiking procedures applied in recovery experiments.

### 3.2. Sample Collection and Preparation

Between May and August 2025, a total of 905 gherkin samples were collected from five major production regions across Türkiye, including Afyonkarahisar (n = 280), Balıkesir (n = 104), İzmir (n = 152), Manisa (n = 214), and Mardin (n = 155) (Figure 5). Approximately 2 kg of each sample were randomly collected at the production stage to ensure representativeness of gherkins intended for retail-level consumer exposure.

**Figure 9:**
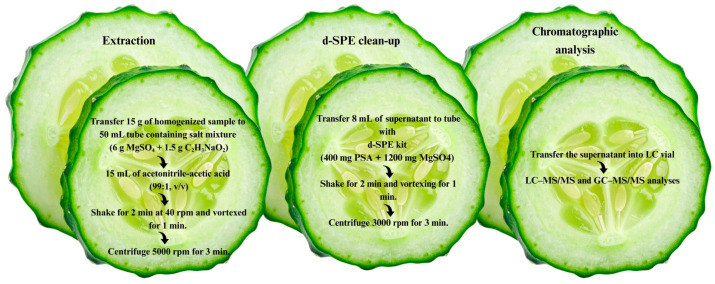
Schematic representation of the QuEChERS-based workflow for sample preparation in pesticide residue analysis of gherkin samples.

To avoid potential losses of surface-associated pesticide residues, no washing or rinsing steps were applied prior to analysis. Samples were homogenised using a laboratory-scale homogeniser (Retsch GM 300, Haan, Germany) and subsequently subjected to pesticide extraction following the QuEChERS method in accordance with AOAC Official Method 2007.01 [31]. The extraction procedure comprised a liquid–liquid microextraction step, followed by a dispersive solid-phase extraction (d-SPE) clean-up. Briefly, 15 g of homogenised sample was weighed into a 50 mL centrifuge tube containing 6 g of anhydrous magnesium sulfate (MgSO_4_) and 1.5 g of sodium acetate (C_2_H_3_NaO_2_). Subsequently, 15 mL of acetonitrile containing 1% (*v*/*v*) acetic acid was added. The mixture was shaken for 2 min using a Multi RS-60 shaker (Biosan Ltd., Riga, Latvia) at 40 rpm, followed by vortex mixing for 1 min, and centrifuged at 5000 rpm for 3 min to achieve phase separation. An 8 mL aliquot of the supernatant was transferred to a 15 mL centrifuge tube containing 400 mg of primary secondary amine (PSA) and 1200 mg of anhydrous MgSO_4_. The extract was mixed for 2 min using a shaker, vortexed for 1 min, and subsequently centrifuged at 3000 rpm for 3 min. The cleaned extract was then filtered through 0.45 μm syringe filter and collected in autosampler vial for instrumental analysis. A schematic overview of the sample preparation workflow is presented in Figure 9.

### 3.3. Chromatographic Analysis

#### 3.3.1. LC-MS/MS Analysis

The determination of 190 target pesticide residues in gherkin samples was carried out using LC-MS/MS. The analytical system consisted of an Agilent Infinity 1260 liquid chromatograph interfaced with an Agilent 6475 triple quadrupole mass spectrometer (Agilent Technologies, Santa Clara, CA, USA). Chromatographic separation was achieved on an Agilent InfinityLab Poroshell 120 EC-C18 column (150 mm × 3.0 mm, 2.7 µm particle size), maintained at 40 °C. The mobile phase comprised (A) water containing 5 mM ammonium formate and (B) methanol. The flow rate was set at 0.4 mL min^−1^, and the injection volume was 5 µL. The autosampler was maintained at 5 °C throughout the analysis. The gradient program was as follows: 0.0–0.25 min, 5% B; 0.25–2.0 min, linear increase to 40% B; 2.0–13.0 min, linear increase to 95% B; 13.0–15.5 min, held at 95% B; 15.5–17.0 min, returned to 5% B for re-equilibration.

Mass spectrometric detection was conducted using an Agilent Jet Stream (AJS) electrospray ionization source operated in both positive and negative ionization modes (ESI^+^/ESI^−^). Capillary voltages were set at 3500 V for positive mode and 3000 V for negative mode. The drying gas temperature and flow rate were maintained at 200 °C and 11.0 L min^−1^, respectively, while sheath gas parameters were set to 350 °C and 12.0 L min^−1^. The nebulizer pressure was adjusted to 35 psi. Compound identification and quantification were conducted in multiple reaction monitoring (MRM) mode, with two transitions monitored for each analyte in accordance with SANTE 11312/2021 v2 guidelines [20]. All MRM transitions and collision energies (CEs) for LC-amenable pesticides are provided in Table S4. Instrument control and data acquisition were performed using Agilent MassHunter Acquisition software (Version 12.2) while quantitative analysis was carried out using MassHunter Quantitative Analysis software (Version 12.1, Agilent Technologies, USA).

#### 3.3.2. GC-MS/MS Analysis

The determination of 20 GC-amenable pesticide residues was performed using a GC–MS/MS system consisting of an Agilent 7010D GC-TQ triple quadrupole mass spectrometer coupled to an Agilent gas chromatograph (Agilent Technologies, Santa Clara, CA, USA). Chromatographic separation was achieved on an HP-5MS capillary column (15 m × 0.25 mm i.d., 0.25 µm, Agilent Technologies). Helium (99.99% purity) was used as the carrier gas at a constant flow rate of 1.0 mL min^−1^. Sample injections were performed in splitless mode with an injection volume of 1 µL, and the splitless valve was maintained closed for 1.5 min. The GC oven temperature program was as follows: 80 °C (1 min), ramped at 20 °C min^−1^ to 170 °C, followed by a ramp at 20 °C min^−1^ to 310 °C (held for 5 min).

Mass spectrometric detection was carried out using electron ionization (EI) operated at 35 eV. The transfer line, ion source, and quadrupole temperatures were maintained at 300 °C, 230 °C, and 150 °C, respectively. Nitrogen was used as the collision gas, whereas helium served as the quench gas. The collision gas flow rate was maintained at 1.5 mL min^−1^ and the quench gas flow rate at 2.35 mL min^−1^. Data acquisition was performed in MRM mode. Detailed MRM transitions and CEs for all GC-amenable pesticides are provided in Table S4. Instrument control, data acquisition, and data processing were conducted using MassHunter GC–TQ software (Version 13.0.324.0, Agilent Technologies, USA).

### 3.4. Method Optimisation and Validation

A total of 210 pesticide residues were included within the analytical scope of the present study, of which 190 pesticides were determined by LC-MS/MS and 20 by GC-MS/MS. The optimisation of LC–MS/MS parameters was carried out through direct infusion of individual pesticide standard solutions (100 μg kg^−1^) into the mass spectrometer using a syringe pump at a flow rate of 10 μL min^−1^. The ionization behaviour of each analyte was initially evaluated using an ESI source operating in both positive and negative modes in order to identify the ionization polarity providing the highest signal response. Subsequently, precursor ions were identified under full-scan acquisition, followed by product-ion scans to investigate the fragmentation behaviour of each compound. Based on the obtained spectra, characteristic precursor-product ion transitions were selected for MRM analysis. Collision energies (CEs) were systematically optimised for each transition to maximize analytical sensitivity and signal stability. Automatic optimization of dwell time was applied during MRM acquisition, resulting in a dwell time of 10 ms per transition to ensure sufficient analytical sensitivity and an adequate number of data points across chromatographic peaks in multi-residue acquisition.

For GC-amenable pesticides, optimisation was performed using EI full-scan analyses to identify the most abundant fragment ions. Product-ion scans were subsequently carried out, and suitable precursor-product ion transitions were selected for MRM acquisition. CEs were optimised to achieve maximum selectivity and analytical sensitivity.

Two MRM transitions per analyte were monitored in compliance with SANTE 11312/2021 v2 guidelines [20]. For data processing and analyte confirmation, retention time tolerances of ±0.1 min and ion ratio tolerances of ±30% were applied, in accordance with the identification criteria specified in the SANTE guidelines.

The analytical performance of the proposed QuEChERS–LC-MS/MS and GC-MS/MS methods was evaluated in the high-water-content matrix in accordance with the SANTE 11312/2021 v2 guidelines for pesticide residue analysis [20]. The validation procedure covered key performance parameters, including selectivity, linearity, LOQs, trueness (recovery), precision, and measurement uncertainty. Method selectivity was assessed through the analysis of more than twenty blank gherkin samples collected over six months. No interfering peaks were observed at the retention times corresponding to the target analytes, confirming the adequate selectivity of the proposed analytical approach. Linearity was evaluated using matrix-matched calibration curves prepared at five concentration levels (0.005, 0.01, 0.05, 0.10, and 0.25 mg kg^−1^). Since certified reference materials were not available for pesticide residues in the gherkin matrix, trueness and precision were assessed using recovery experiments based on fortified blank samples. Homogenised matrix samples were spiked with pesticide standard mixtures at two concentration levels (0.01 and 0.05 mg kg^−1^). Five replicate samples (n = 5) were prepared and analysed at each fortification level. Method repeatability was evaluated based on the RSDr% obtained from recovery experiments performed under the same operating conditions on the same day. The RSD_WR_% was assessed over five days by two independent laboratory analysts performing sample fortification, extraction, and analysis on different days. The LOQs were calculated as 10 times the standard deviation of the replicate analyses (n = 10) of blank samples at the low fortification level, which could be quantified with acceptable recoveries (between 70 and 120%) and repeatability (RSDr ≤ 20%). Measurement uncertainty was estimated for each analyte by combining the contributions of trueness (bias) and within-laboratory reproducibility. The expanded uncertainty was expressed using a coverage factor of *k* = 2, corresponding to an approximate 95% confidence level, in accordance with the SANTE guidelines [20]. Procedural blanks, duplicate samples, and spiked quality control samples were included periodically within analytical batches to monitor method performance.

### 3.5. Integrated Quality Assessment Based on the *IqR*

The IqR was employed to evaluate the overall quality of gherkin samples with respect to the combined occurrence of multiple pesticide residues across five major production regions in Türkiye. For each sample, the IqR value was calculated as the sum of the ratios between the measured pesticide residue concentrations (*PRC*) and their corresponding MRLs, as expressed in Equation (1):

(1) $$IqR=\sum_{i=0}^{n}\frac{PRCi}{MRLi}$$

where *i* represents an individual pesticide residue detected in a given sample, and *n* corresponds to the total number of pesticide residues identified.

Based on the resulting IqR values, gherkin samples were classified into four quality categories, namely excellent (IqR = 0), good (0 < IqR ≤ 0.6), acceptable (0.6 < IqR ≤ 1.0), and unacceptable (IqR > 1.0), in accordance with the classification scheme proposed by Mac Loughlin et al. [30]. This integrative metric enables a holistic evaluation of sample quality by simultaneously considering both the occurrence and the relative magnitude of multiple pesticide residues in relation to their regulatory thresholds, thereby providing a comprehensive measure of overall residue-related quality.

### 3.6. Health Risk Assessment

#### 3.6.1. Risk Ranking

Pesticide risk scores were calculated using a matrix-based risk ranking system originally developed by the UK’s Veterinary Residues Committee [32], which integrates toxicity- and exposure-related attributes to prioritize pesticide residues detected in gherkin samples. The composite risk score (S) was derived from six components: acute toxicity (A), toxic potency (B), dietary contribution (C), application frequency (D), potential exposure of vulnerable population groups (E), and residue levels relative to the MRLs (F). Each component was scored using the established criteria of the original risk ranking matrix (Table S5), and the assigned scores for indices A-F are presented in Table S6. The overall risk scores were calculated using Equation (2):

(2) $$S=\left(A+B\right)\times \;\left(C+D+\;E\right)\;\times \;F$$

where A represents acute toxicity based on median lethal dose (*LD*_50_) values [33], B denotes toxic potency derived from ADI values [34], C corresponds to the dietary contribution of gherkins based on consumption data [35], D reflects the frequency of pesticide application relative to the crop growth period, E is set to 3 due to insufficient evidence regarding high-exposure population groups, and F is derived from measured residue levels in comparison with the corresponding MRLs.

The frequency of pesticide application during crop development was expressed as the frequency of dosing (*FOD*), calculated as the ratio between the number of pesticide applications (*N*) and the duration of the gherkin growth period (*P*, days), as shown in Equation (3):

(3) $$FOD=\;\frac{N}{P}\;\times \;100$$

The residue level score (*F*) was calculated based on the distribution of measured residue concentrations relative to the corresponding MRLs using the following equation (Equation (4)):

(4) $$F=\frac{\left(F_{0}\;\times \;1)\;+\;{(F}_{1}\times \;2)\;+\;{(F}_{2}\;\times \;3)+\;{(F}_{3}\;\times \;4\right)}{n}$$

where F_0_ represents the number of gherkin samples with no detectable pesticide residues, F_1_ denotes the number of samples with residue levels below the MRL, F_2_ corresponds to samples with residue levels between ≥1 and 10 times the MRL, F_3_ refers to samples exceeding ≥10 times the MRL, and *n* is the total number of analysed samples.

#### 3.6.2. Deterministic Chronic and Acute Dietary Exposure and Risk Assessment

A deterministic dietary exposure assessment was conducted to estimate the potential chronic and acute health risks associated with pesticide residues detected in gherkin samples. Exposure calculations were performed separately for adults and children (3–10 years), and the national estimated daily intake (NEDI, mg kg^−1^ bw day^−1^) was calculated using Equation (5):

(5) $$\mathrm{NEDI}=\;\frac{\mathrm{Residue}\;\mathrm{concentration}\;\left(\mathrm{mg}/\mathrm{kg}\right)\;\times \;\mathrm{consumption}\;\mathrm{rate}\;(\mathrm{kg})}{\mathrm{Body}\;\mathrm{weight}\;(\mathrm{kg})}$$

Left-censored data (residue concentrations below the LOQ) were recognised as a major source of uncertainty in exposure estimation. In accordance with EFSA guidance [36], a substitution approach was applied for uncertainty characterization, whereby non-detects were replaced with zero (LB), LOQ/2 (middle bound, MB), and LOQ (UB). Daily per capita gherkin consumption was assumed to be 0.0011 kg for adults and 0.0004 kg for children, based on EFSA consumption data [35]. Standard body weights of 70 kg for adults and 23 kg for children were applied [37].

The *HQc* for each pesticide was subsequently calculated as the ratio of the NEDI to the corresponding *ADI*, as expressed in Equation (6):

(6) $$HQc\;=\;\frac{NEDI}{ADI}$$

Acute (short-term) dietary exposure was assessed using the International Estimated Short-Term Intake (IESTI, mg kg^−1^ bw), calculated according to the WHO/FAO [38] Case 2a model, which is applicable when the unit weight of the edible portion satisfies 25 g ≤ *Ue* < *LP*. The IESTI was calculated using Equation (7):

(7) $$IESTI=\;\frac{Ue\;\times \;HR\;\times \;v\;+\left(LP\;-Ue\right)\;\times \;HR}{bw}$$

where *Ue* represents the unit weight of the edible portion (0.0544 kg for gherkins), *HR* is the highest residue detected in the composite sample (mg kg^−1^), *v* is the variability factor set to 3, and *LP* denotes the large portion size (0.168 kg for adults and 0.092 kg for children) [39].

The *HQa* was calculated as the ratio of IESTI to ARfD, as shown in Equation (8):

(8) $$HQa=\;\frac{IESTI}{ARfD}$$

For pesticides lacking established ARfD values, the ADI was conservatively applied for the calculation of *HQa*. ARfD and ADI values for individual pesticides were obtained from the EU Pesticides Database [34].

Cumulative chronic and acute risks were expressed using the *HI* approach, obtained by summing individual *HQ* values across all detected pesticides, as shown in Equation (9):

(9) HI=Σ HQ

Health risks were considered acceptable when individual *HQc*, *HQa*, or cumulative risk indices (*HIc* and *HIa*) values remained below unity (<1), indicating that estimated exposures did not exceed the corresponding toxicological reference values. Values exceeding this threshold were interpreted as indicative of a potential increase in health concern related to pesticide exposure.

#### 3.6.3. Probabilistic Risk Assessment by Monte Carlo Simulation

A probabilistic risk assessment was conducted using the MCS approach to capture variability and uncertainty in dietary exposure estimates and to provide a more realistic characterization of potential consumer health risks. The simulation was implemented in Microsoft Excel integrated with Oracle Crystal Ball^®^ software (version 11.1.34190, Oracle, Austin, TX, USA), with 10,000 iterations performed for each exposure scenario. The log-normal distribution was assumed for residue concentrations and consumption data due to their right-skewed nature, whereas body weight was modelled using a normal distribution. For each pesticide detected in gherkin samples, probability distributions were generated to characterize chronic dietary exposure, allowing the probability of exceeding the corresponding ADI thresholds to be estimated. The probabilistic framework incorporated variability in residue concentrations, consumption patterns, and body weights, thereby providing a more realistic and robust characterization of potential health risks than point-estimate–based approaches.

In addition, SA was carried out within the MCS framework to quantify the relative contribution of individual input variables to the overall variability in the *HIc*. Regression-based sensitivity measures were applied to identify the key parameters influencing the variance of the model output and to determine the primary drivers of cumulative dietary exposure and associated health risks.

## 4. Conclusions

An integrated QuEChERS-LC-MS/MS and GC-MS/MS workflow was successfully applied for the multi-residue analysis of pesticides in gherkins, with satisfactory method performance criteria specified in the SANTE 11312/2021 v2 guidelines. Widespread pesticide occurrence, including multi-residue contamination and occasional MRL exceedances, was observed, indicating variability in pesticide management across production regions. Deterministic and probabilistic risk assessments indicated that chronic dietary exposure to pesticide residues through gherkin consumption is unlikely to pose significant health risks, although certain acute exposure scenarios for children warrant continued monitoring. The study is subject to several limitations, including seasonal sampling conducted exclusively between June and August 2025 and the use of unwashed samples, which may not fully reflect typical consumer practices. In addition, the analytical scope may not fully cover certain highly polar pesticide residues (e.g., glyphosate and related compounds), which may influence the overall residue profile. These findings highlight the value of the validated analytical workflow for the large-scale monitoring of pesticide residues in cucurbit crops, supporting evidence-based regulatory decision-making and emphasising the need for ongoing surveillance and targeted risk management strategies.

## Data Availability

The original contributions presented in the study are included in the article and Supplementary Material; further inquiries can be directed to the corresponding authors.

## Supplementary Materials

The following supporting information can be downloaded at https://www.mdpi.com/article/10.3390/molecules31081248/s1. Figure S1: Probabilistic risk assessment of gherkin pesticide residues using *MCS*, showing *HIc* distributions for adults and children, with SA highlighting the main contributors to risk variability; Table S1: In-house validation data for 210 pesticide residues in gherkin samples by LC-MS/MS and GC-MS/MS; Table S2: Residue levels of 37 pesticides detected in gherkin samples; Table S3: Chronic and acute hazard quotients (*HQc* and *HQa*) for pesticides detected in gherkin samples for adults and children; Table S4: MS/MS parameters (precursor ions, product ions, and collision energies) for target pesticides analysed by LC-MS/MS and GC-MS/MS; Table S5: Definition and individual scores of indices for the pesticide residual risk ranking; Table S6: Assigned scores for indices A-F used in the pesticide residue risk scoring system.
