# Supplementary material for: High-Throughput Determination of 210 Pesticide Residues in Gherkins by QuEChERS Coupled with LC-MS/MS and GC-MS/MS
Source: Molecules. 2026 Apr 9;31(8):1248. doi: 10.3390/molecules31081248 (PMC13118492; doi:10.3390/molecules31081248)
Supplement: Supplementary file 1 [file molecules-31-01248-s001.zip › molecules-4234748-supplementary.pdf]

High-Throughput Determination of 210 Pesticide Residues in Gherkins by QuEChERS Coupled with LC-MS/MS and GC-MS/MS

Mehmet Keklik [1], Eylem Odabas [2], Tuba Buyuksirit-Bedir [2], Ozgur Golge [3], Miguel Ángel González-Curbelo [4,*] and Bulent Kabak [2,*]

[1] Food Control Laboratory, Yonca Gıda, Şehzadeler, Manisa 45030, Türkiye; mkeklik@yildiz.edu.tr
[2] Department of Food Engineering, Faculty of Engineering and Natural Sciences, Hitit University, Corum 19030, Türkiye
[3] Department of Gastronomy and Culinary Arts, Faculty of Tourism, Alanya Alaaddin Keykubat University, Alanya 07425, Türkiye
[4] Departamento de Ciencias Básicas, Facultad de Ingeniería, Universidad EAN, Calle 79 n11-45, Bogotá 110221, Colombia
* Correspondence: magonzalez@universidadean.edu.co (M.Á.G.-C.); bulentkabak@hitit.edu.tr (B.K.)

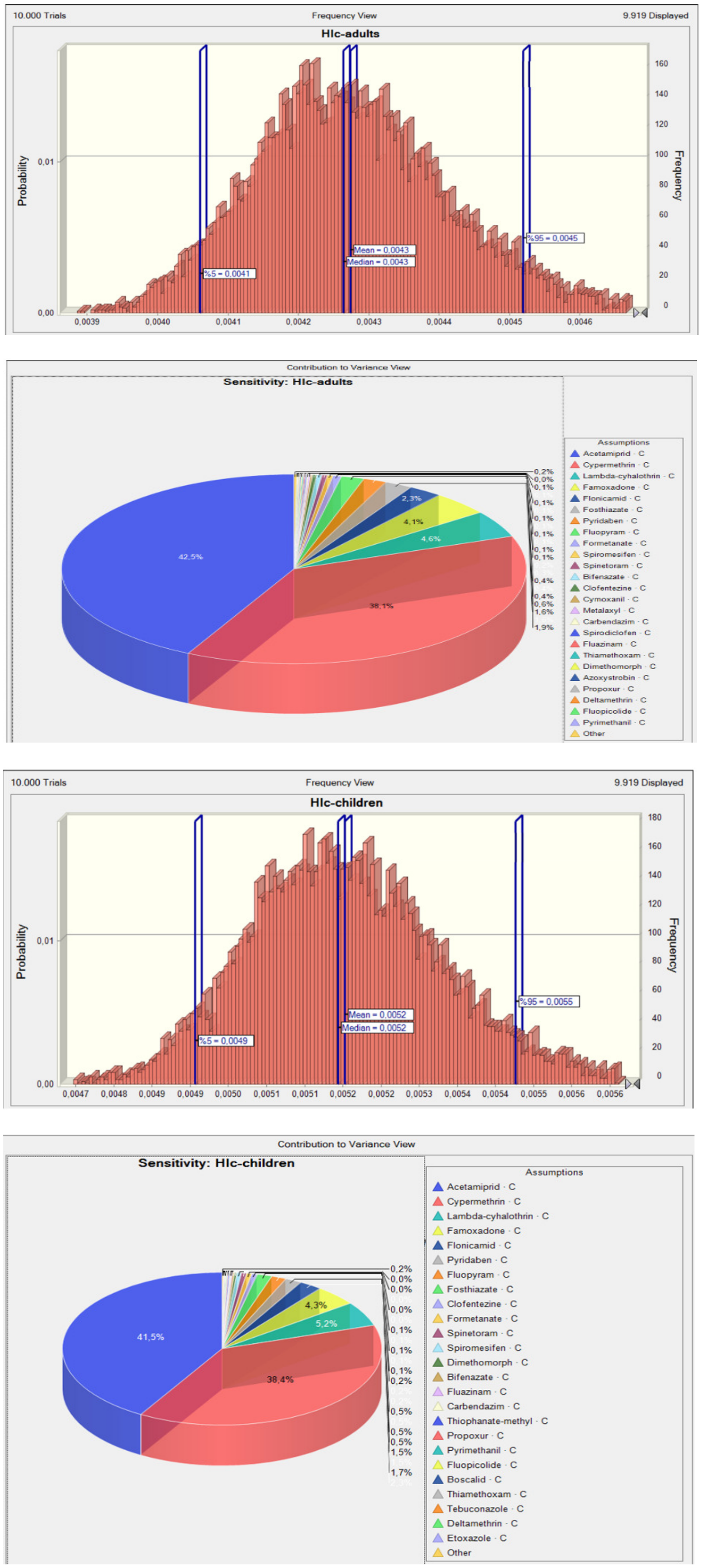

Figure S1. Probabilistic risk assessment of gherkin pesticide residues using *MCS*, showing *HIc* distributions for adults and children, with SA highlighting the main contributors to risk variability.

Table S1. In-house validation data for 210 pesticide residues in gherkin samples by LC-MS/MS and GC-MS/MS.

**Pesticide**	**LOQ (mg kg^{-1})**	**Recovery (%)**		**Repeatability (%RSD$_r$, *n*=5)**		**Within-laboratory reproducibility (%RSD$_R$, *n*=10)**		***U* (%)**
		0.01 mg kg^{-1}	0.05 mg kg^{-1}	0.01 mg kg^{-1}	0.05 mg kg^{-1}	0.01 mg kg^{-1}	0.05 mg kg^{-1}	
2,4-D	0.01	97.0	93.7	4.69	2.80	5.52	5.45	14.4
2-phenylphenol	0.01	91.4	93.9	6.91	4.61	5.27	5.52	18.2
Acephate*	0.01	91.2	95.2	5.29	4.10	14.33	6.07	24.5
Acequinocyl	0.01	92.0	101.3	4.81	2.61	6.57	10.44	18.3
Acetamiprid	0.01	95.2	99.4	3.46	3.50	4.88	7.72	13.7
Acibenzolar-s-methyl*	0.01	92.2	97.2	4.65	2.85	5.43	4.22	14.3
Alachlor*	0.01	84.2	93.1	3.63	5.67	11.78	6.97	29.4
Aldicarb*	0.01	89.2	94.6	5.81	4.12	4.68	9.88	21.8
Aldicarb sulfone*	0.01	98.6	99.6	5.53	4.52	10.91	11.75	22.7
Aldicarb sulfoxide*	0.01	92.4	96.3	5.57	5.56	8.84	6.92	19.4
Ametoctradin	0.01	91.6	92.7	5.52	4.14	8.58	6.79	22.0
Amitraz*	0.01	86.2	84.7	16.20	9.17	5.56	11.53	33.7
Atrazine*	0.01	94.0	91.2	4.60	4.84	7.18	4.63	18.9
Azinphos-methyl*	0.01	84.8	91.0	5.80	5.57	9.61	8.18	30.0
Azoxystrobin	0.01	96.0	98.8	4.50	5.83	5.79	6.42	13.3
Benfuracarb*	0.01	97.8	89.2	6.50	7.31	12.88	9.83	26.2
Bensulfuron-methyl	0.01	93.0	95.8	6.51	5.04	12.18	3.90	19.6
Bentazone	0.01	90.0	93.0	6.02	3.80	8.13	5.30	21.7
Bifenazate	0.01	113.1	112.2	4.08	4.28	6.33	11.04	30.7
Bifenthrin*	0.01	91.0	112.7	7.99	3.82	9.36	19.76	29.4
Boscalid	0.01	91.2	98.0	4.98	2.99	7.22	4.65	16.0
Bromoxynil*	0.01	80.9	82.3	8.31	5.42	9.41	8.37	40.9
Bromuconazole	0.01	92.0	97.2	4.23	2.99	5.10	7.16	16.3
Bupirimate	0.01	92.8	98.5	4.89	3.86	5.73	2.85	12.2
Buprofezin	0.01	87.4	92.0	5.51	3.86	12.02	9.43	29.7
Cadusafos*	0.01	90.6	91.0	3.61	3.40	7.96	4.49	22.2
Captan	0.01	77.3	105.8	4.97	8.44	6.16	9.89	23.3
Carbaryl*	0.01	90.0	92.3	4.68	2.53	6.94	6.05	22.0
Carbendazim*	0.01	97.4	102.0	4.84	3.39	7.48	3.48	11.0
Carbofuran*	0.01	94.4	104.8	5.27	2.36	4.06	12.41	16.5
Carbosulfan*	0.01	91.4	110.5	5.27	3.09	8.13	10.94	19.2
Carboxin*	0.01	94.8	97.3	4.47	3.62	3.06	5.44	11.6
Chlorfluazuron*	0.01	88.2	93.5	9.22	5.24	12.03	9.66	28.4
Chloridazon*	0.01	94.2	97.4	3.93	2.88	7.72	7.31	17.2
Chlorpyrifos*	0.01	88.8	87.3	5.06	6.76	6.81	5.04	26.7
Chlorpyrifos-methyl*	0.01	101.4	100.5	7.48	7.70	8.65	7.93	16.7
Chlorsulfuron*	0.01	84.2	97.1	6.08	7.70	9.27	6.12	24.2
Clethodim	0.01	89.4	83.7	5.78	3.43	11.21	10.00	34.3
Clodinafop	0.01	80.4	91.8	4.20	3.20	3.97	4.29	29.0
Clofentezine*	0.01	96.2	94.7	6.23	4.25	5.87	8.79	17.3
Clothianidin*	0.01	88.6	95.0	6.21	2.70	5.96	7.13	21.0
Cyazofamid	0.01	96.4	98.9	6.88	3.47	8.02	2.51	11.5
Cycloate*	0.01	90.2	84.7	7.35	4.23	8.44	10.47	31.4
Cyflumetofen	0.01	89.4	97.0	4.95	3.48	16.32	4.96	25.3
Cymoxanil	0.01	97.4	100.9	4.75	3.14	5.75	5.51	11.4
Cypermethrin	0.01	81.2	84.4	5.70	7.50	11.78	8.85	40.1
Cyproconazole*	0.01	92.4	97.8	3.51	4.36	4.92	5.57	14.4
Cyprodinil	0.01	96.8	95.6	3.51	4.36	5.44	8.11	15.5

Dazomet	0.01	86.4	99.1	5.01	3.86	4.36	3.77	16.6
Deltamethrin	0.01	95.0	98.4	4.12	5.35	8.66	8.57	18.5
Demeton-S-methyl*	0.01	93.6	98.0	3.31	5.46	6.85	3.39	13.2
Demeton-S-methyl- sulfone*	0.01	90.4	92.3	5.97	2.64	7.17	11.49	25.4
Demeton-S-methyl- sulfoxide*	0.01	93.4	82.8	4.85	10.89	3.37	10.89	27.7
Desmedipham*	0.01	93.0	96.8	3.83	3.18	12.53	3.00	18.6
Diafenthiuron*	0.01	91.2	96.1	6.22	2.05	11.67	3.18	19.5
Diazinon*	0.01	90.3	87.8	5.87	3.71	7.88	9.95	28.2
Dichlofluanid*	0.01	91.0	99.8	6.15	4.14	6.87	10.38	19.6
Dichlorvos*	0.01	92.6	95.8	4.44	2.78	4.56	4.93	15.0
Dicrotophos*	0.01	90.2	91.8	5.38	3.05	6.72	5.19	21.6
Diethofencarb*	0.01	93.0	97.6	4.09	4.47	6.16	5.45	14.9
Difenoconazole	0.01	96.8	100.6	13.92	4.59	9.56	7.22	17.0
Diflubenzuron*	0.01	86.8	100.1	10.49	5.62	11.64	4.41	20.7
Dimethenamid*	0.01	92.0	94.8	3.24	2.74	8.24	4.27	18.2
Dimethoate*	0.01	99.6	96.0	5.17	3.02	5.06	9.23	15.0
Dimethomorph*	0.01	88.8	95.3	6.30	3.85	8.78	7.56	22.8
Dinocap*	0.01	96.4	98.3	6.40	3.97	7.37	7.26	15.6
Dinotefuran*	0.01	106.2	97.1	4.74	6.25	8.32	8.69	17.3
Diuron*	0.01	95.6	95.1	3.53	5.34	2.99	7.57	14.1
Dodine	0.01	92.0	92.2	7.39	4.22	9.07	6.99	22.5
Epoxiconazole*	0.01	91.0	99.7	6.06	4.00	10.22	2.67	15.9
Ethiofencarb*	0.01	100.2	91.8	5.28	4.85	7.41	4.85	14.6
Ethofumesate	0.01	91.6	98.0	4.34	3.98	9.63	4.47	17.5
Etofenprox	0.01	91.8	98.6	3.62	3.47	8.84	5.83	17.5
Etoxazole	0.01	88.0	95.6	4.67	3.43	8.95	4.78	21.4
Famoxadone*	0.01	96.4	101.2	5.60	5.00	9.50	10.10	19.7
Fenamidone*	0.01	92.6	95.5	5.10	3.68	5.94	5.97	16.8
Fenarimol*	0.01	93.2	93.2	4.07	6.87	11.05	4.47	20.6
Fenazaquin	0.01	89.4	94.1	6.59	2.52	9.23	3.46	20.8
Fenbuconazole*	0.01	95.4	98.9	5.93	2.45	6.93	8.80	16.7
Fenhexamid	0.01	94.0	97.6	6.26	3.49	6.92	2.67	12.7
Fenoxaprop-P-ethyl	0.01	90.4	96.4	5.30	3.54	11.69	7.67	23.4
Fenoxycarb*	0.01	93.2	98.2	7.58	3.60	7.26	6.43	16.2
Fenpropathrin*	0.01	91.8	96.8	4.76	4.73	7.90	5.87	17.9
Fenpyroximate	0.01	89.4	96.0	6.24	4.44	9.76	6.23	21.7
Fenthion*	0.01	88.8	96.9	6.02	3.24	5.27	4.36	17.2
Flonicamid	0.01	87.2	92.3	6.94	3.34	4.69	6.22	23.2
Fluazinam	0.01	90.2	93.2	4.61	5.15	7.32	3.93	20.1
Fludioxonil	0.01	93.4	97.7	4.41	3.64	6.30	2.32	12.4
Flufenoxuron*	0.01	86.2	90.9	7.61	3.77	7.28	6.66	26.8
Fluopicolide	0.01	92.0	96.3	5.61	3.23	5.66	5.27	16.0
Fluopyram	0.01	104.2	92.3	4.99	6.91	13.69	6.91	20.9
Flutriafol*	0.01	91.6	101.4	8.78	6.59	5.23	6.73	13.9
Fonofos*	0.01	84.0	92.9	6.35	3.33	15.54	2.90	29.6
Formetanate	0.01	83.8	97.4	7.33	4.30	11.24	4.65	24.6
Fosthiazate	0.01	95.4	95.6	4.85	2.80	5.12	4.72	13.3
Furathiocarb*	0.01	90.8	94.6	6.90	4.76	9.91	6.67	22.1
Haloxyfop*	0.01	91.0	96.7	6.15	3.54	15.52	6.84	25.5
Haloxyfop-P-methyl*	0.01	90.2	97.6	5.58	3.39	8.71	6.68	19.6
Heptenophos*	0.01	91.6	96.7	3.68	2.53	6.52	4.56	16.1
Hexaconazole*	0.01	95.4	94.8	5.14	3.77	11.02	7.05	20.6
Hexaflumuron*	0.01	89.8	101.2	5.14	3.77	17.48	9.99	28.9
Hexythiazox	0.01	93.0	95.9	6.82	2.81	7.28	7.06	18.1
Imazalil	0.01	91.0	93.6	6.81	2.81	7.61	3.81	19.2

Imidacloprid*	0.01	99.2	100.1	7.56	4.53	11.47	10.21	21.7
Indoxacarb*	0.01	91.4	93.9	8.82	5.30	8.18	5.83	20.3
Iodosulfuron	0.01	83.8	91.3	6.60	3.34	8.10	7.90	29.6
Ioxynil*	0.01	94.0	89.9	10.52	9.02	8.14	10.16	24.4
Iprodione*	0.01	91.2	79.3	8.43	5.68	13.33	15.56	41.3
Iprovalicarb	0.01	87.0	96.1	4.88	3.73	6.79	4.51	20.3
Kresoxim-methyl	0.01	92.8	98.9	6.75	5.04	8.27	5.34	15.9
Lambda-cyhalothrin	0.01	113.5	81.9	3.15	6.28	14.96	6.53	22.0
Lenacil	0.01	93.2	95.9	4.32	3.66	5.11	3.30	13.8
Linuron*	0.01	93.4	98.5	5.05	4.04	4.25	5.06	12.3
Lufenuron*	0.01	91.6	95.1	8.48	5.23	9.47	8.74	22.5
Malathion	0.01	92.0	97.9	3.40	2.67	7.12	4.20	15.2
Malaoxon	0.01	96.6	95.0	4.58	3.23	6.65	5.53	14.8
Mandipropamid	0.01	94.0	99.4	4.49	2.49	4.88	4.69	11.6
Mecarbam*	0.01	88.2	94.9	5.18	3.64	8.31	3.17	20.4
Metalaxyl	0.01	97.1	108.2	10.11	6.49	8.78	4.89	14.7
Metamitron	0.01	87.6	100.0	3.54	2.69	6.81	5.00	17.1
Methacrifos*	0.01	95.2	94.6	5.07	3.30	7.76	6.25	17.3
Methamidophos*	0.01	93.6	96.7	6.27	3.30	11.55	5.31	19.5
Methiocarb*	0.01	94.6	95.3	3.99	3.23	4.66	1.92	12.1
Methomyl*	0.01	87.6	88.4	6.26	5.98	9.21	5.98	28.4
Metolachlor*	0.01	80.8	90.4	6.87	2.73	5.81	7.42	31.7
Metribuzin*	0.01	91.6	96.9	5.89	4.61	5.35	6.52	16.5
Mevinphos*	0.01	84.2	91.9	9.51	6.43	9.77	4.82	28.0
Monocrotophos*	0.01	89.4	89.3	4.48	3.14	6.31	4.25	23.8
Monolinuron*	0.01	83.6	98.8	5.84	3.83	4.89	4.17	19.8
Myclobutanil*	0.01	97.2	96.6	7.46	2.96	5.74	6.78	14.0
Nicosulfuron	0.01	80.6	93.0	6.62	3.52	11.87	6.79	32.3
Nuarimol*	0.01	85.0	98.1	7.04	4.79	11.86	5.98	24.6
Omethoate*	0.01	94.6	96.9	6.31	5.30	14.65	5.59	22.0
Oxadixyl*	0.01	91.4	94.1	5.56	3.02	4.67	6.38	18.2
Oxamyl*	0.01	94.0	96.6	5.67	4.00	7.85	3.40	14.7
Oxyfluorfen	0.01	110.8	75.7	7.14	4.33	3.38	6.50	16.7
Paraoxon-ethyl	0.01	99.4	99.6	6.08	4.53	6.02	4.82	10.9
Penconazole	0.01	95.4	98.6	6.92	2.68	9.02	5.58	15.8
Pendimethalin	0.01	92.4	94.2	6.27	3.12	8.79	6.09	20.0
Phenmedipham	0.01	85.2	96.5	9.14	4.03	10.68	6.61	25.2
Phenthoate*	0.01	90.8	98.2	3.47	3.99	8.12	8.26	19.7
Phorate*	0.01	103.6	96.5	6.81	11.22	7.67	9.62	17.3
Phosalone*	0.01	97.4	96.3	4.55	3.64	3.11	5.03	10.3
Phosphamidon*	0.01	90.8	97.5	4.89	2.48	5.10	9.42	18.6
Phosmet*	0.01	96.2	98.1	4.75	2.39	6.39	4.00	11.9
Pirimicarb	0.01	88.0	96.0	5.36	3.57	3.79	4.74	18.1
Pirimiphos-ethyl*	0.01	92.4	79.4	5.23	4.03	7.81	10.40	33.6
Pirimiphos-methyl	0.01	86.9	91.1	5.78	3.72	7.10	7.87	26.6
Prochloraz*	0.01	94.8	93.9	4.36	3.64	8.26	6.10	18.3
Profenofos*	0.01	106.1	110.1	8.43	3.41	17.66	5.52	28.3
Profoxydim*	0.01	87.0	84.9	7.29	4.34	10.45	9.86	34.7
Promecarb*	0.01	96.4	99.5	6.90	4.97	5.34	3.58	9.8
Prometryn*	0.01	88.4	97.2	4.50	2.44	6.29	3.59	17.5
Propamocarb	0.01	88.4	93.4	3.96	2.94	4.26	2.94	19.6
Propaquizafop	0.01	87.6	96.3	5.25	3.44	14.33	5.09	25.2
Propargite*	0.01	92.4	95.7	6.68	3.51	6.97	5.95	17.6
Propazine*	0.01	97.2	99.1	5.06	2.35	7.46	4.22	12.3
Propiconazole*	0.01	98.6	101.1	5.33	4.73	6.37	6.57	12.9

Propoxur*	0.01	94.8	97.9	5.37	3.69	5.66	7.47	15.0
Propyzamide	0.01	96.4	99.7	6.09	2.68	5.88	2.45	9.2
Prosulfuron	0.01	84.8	95.6	6.41	4.71	6.68	7.25	24.0
Prothiofos*	0.01	107.4	89.4	5.16	4.61	7.40	5.83	13.6
Pymetrozine*	0.01	90.0	95.8	7.18	3.25	7.08	3.86	17.9
Pyrazophos*	0.01	113.7	77.7	2.79	3.94	14.08	3.77	19.8
Pyridaben	0.01	89.2	94.4	7.26	3.81	6.11	5.06	19.8
Pyridaphenthion*	0.01	93.4	99.5	6.06	3.40	6.40	5.49	13.8
Pyridate	0.01	87.2	90.6	5.64	3.83	8.17	2.18	24.5
Pyrimethanil	0.01	89.0	93.3	5.73	4.16	5.19	4.31	20.1
Pyriproxyfen	0.01	88.0	95.5	5.63	3.29	4.14	3.42	18.1
Rimsulfuron	0.01	84.0	94.6	9.26	4.34	6.58	4.78	24.2
Sethoxydim*	0.01	92.8	86.1	7.41	3.92	12.12	7.82	29.0
Simazine*	0.01	92.0	97.4	4.91	4.91	10.49	7.32	20.7
Spinosyn A	0.01	94.2	95.3	5.43	3.71	8.28	6.20	17.9
Spinosyn D	0.01	94.0	95.3	6.73	3.27	5.85	5.97	15.9
Spinetoram*	0.01	92.4	93.8	2.85	3.81	8.76	6.97	20.9
Spirodiclofen*	0.01	89.6	93.8	11.61	5.39	6.58	7.33	21.7
Spiromesifen*	0.01	95.1	76.4	6.73	5.15	9.61	11.18	35.3
Spiroxamine*	0.01	89.0	98.9	3.86	3.45	6.88	8.45	19.5
Tau-fluvalinate	0.01	90.1	101.2	5.27	10.65	6.56	9.70	18.4
Tebuconazole	0.01	94.2	98.9	3.93	3.87	2.93	6.42	11.6
Tebufenozide	0.01	97.2	96.9	4.77	4.21	6.55	3.00	11.2
Tebufenpyrad	0.01	89.4	96.8	4.95	4.99	11.94	6.89	23.3
Teflubenzuron*	0.01	91.8	87.9	10.45	3.96	8.84	3.01	23.5
Tepraloxydim*	0.01	87.2	86.8	9.38	3.94	15.19	8.98	35.5
Terbufos*	0.01	94.8	96.4	5.28	4.24	8.03	9.48	19.6
Terbuthylazine	0.01	81.4	96.8	12.67	4.21	7.19	6.62	25.8
Terbuthylazine- desethyl	0.01	100.1	108.7	7.37	3.42	9.58	17.29	28.3
Terbutryn*	0.01	92.8	101.2	6.03	3.07	3.95	2.58	8.9
Tetraconazole	0.01	92.4	97.3	5.93	3.37	7.12	4.43	15.5
Thiabendazole	0.01	90.6	95.6	6.42	2.89	2.94	5.56	16.2
Thiacloprid*	0.01	95.6	97.1	4.04	3.56	4.62	7.41	14.1
Thiamethoxam*	0.01	87.0	92.9	7.45	1.78	8.55	5.12	24.3
Thifensulfuron-methyl	0.01	79.8	92.7	6.19	3.79	11.37	8.36	33.8
Thiobencarb*	0.01	85.6	97.4	7.93	4.08	8.79	7.27	23.4
Thiodicarb	0.01	95.0	101.0	7.47	5.14	2.25	4.17	7.6
Thiophanate-methyl*	0.01	98.0	101.0	5.09	3.17	10.18	3.96	14.2
Tolylfluanid*	0.01	108.4	86.9	4.99	5.38	11.52	6.67	18.8
Tralkoxydim*	0.01	90.0	85.6	9.31	4.06	11.60	7.90	31.2
Triadimefon*	0.01	88.8	99.6	9.27	3.06	9.70	2.47	16.8
Triadimenol*	0.01	95.0	95.8	6.30	3.42	5.07	6.04	14.4
Tri-allate	0.01	89.4	94.3	7.32	4.34	12.19	2.30	21.8
Triasulfuron*	0.01	90.4	99.6	5.79	3.02	10.24	4.88	18.1
Trichlorfon*	0.01	86.4	97.5	4.73	3.30	5.48	5.26	19.4
Trifloxystrobin	0.01	91.2	95.0	3.46	5.97	12.61	9.12	25.7
Triflumizole*	0.01	93.4	95.3	5.62	5.52	7.47	9.01	20.0
Triflumuron*	0.01	101.8	103.4	6.90	3.72	1.78	9.51	12.4
Triticonazole	0.01	90.6	95.1	7.79	4.14	9.99	2.67	19.1
Valifenalate	0.01	104.8	94.7	10.12	5.07	8.55	5.89	14.4
Vamidothion*	0.01	94.4	96.2	3.13	3.19	6.02	8.56	17.3
Vinclozolin*	0.01	103.7	106.5	6.83	4.23	9.57	4.79	17.6

*Not approved in the EU.

Table S2. Residue levels of 37 pesticides detected in gherkin samples.

Pesticide	Pesticide type	EU MRL (mg kg^{-1})	No. of detectable samples (%)	No. of samples >MRLs (%)	Range (mg kg^{-1})	
					Min.-Max.	Mean
Acequinocyl	AC[a]	0.04	1 (0.11)	1 (0.11)	0.100	0.100
Acetamiprid	IN[b]	0.6	120 (13.26)	–	0.015–0.431	0.040
Ametoctradin	FU[c]	3	80 (8.84)	–	0.013–0.570	0.047
Azoxystrobin	FU	1	101 (11.16)	–	0.011–0.447	0.038
Bifenazate	IN	0.01	4 (0.44)	4 (0.44)	0.016–0.048	0.029
Boscalid	FU	4	13 (1.44)	–	0.016–0.106	0.042
Captan	FU	0.03	2 (0.22)	1 (0.11)	0.026–0.058	0.042
Carbendazim*	FU	0.1	5 (0.55)	–	0.014–0.046	0.029
Chlorpyrifos*	IN	0.01	2 (0.22)	2 (0.22)	0.021–0.043	0.032
Clofentezine*	IN/AC	0.2	14 (1.55)	1 (0.11)	0.015–0.250	0.052
Cyazofamid	FU	0.2	3 (0.33)	–	0.018–0.066	0.034
Cyflumetofen	IN/AC	0.4	7 (0.77)	–	0.016–0.160	0.046
Cymoxanil	FU	0.08	3 (0.33)	1 (0.11)	0.024–0.098	0.052
Cypermethrin	IN	0.2	8 (0.88)	2 (0.22)	0.026–0.821	0.215
Deltamethrin	IN	0.2	2 (0.22)	–	0.025–0.087	0.056
Dimethomorph*	FU	0.5	31 (3.43)	–	0.015–0.230	0.032
Etoxazole	IN	0.01	7 (0.77)	7 (0.77)	0.012–0.086	0.030
Famoxadone*	FU	0.01	19 (2.10)	19 (2.10)	0.011–0.084	0.027
Flonicamid	IN	0.5	328 (36.24)	–	0.013–0.382	0.048
Fluazinam	FU	0.01	2 (0.22)	2 (0.22)	0.021–0.040	0.031
Fluopicolide	FU	0.5	23 (2.54)	–	0.015–0.130	0.030
Fluopyram	FU	0.6	33 (3.65)	–	0.015–0.160	0.035
Formetanate	IN	0.3	2 (0.22)	–	0.027–0.068	0.048
Fosthiazate	IN/NE[d]	0.02	3 (0.33)	3 (0.33)	0.021–0.037	0.031
Lambda-cyhalothrin	IN	0.15	7 (0.77)	–	0.025–0.073	0.049
Metalaxyl	FU	0.01	26 (2.87)	26 (2.87)	0.012–0.343	0.035
Propamocarb	FU	5	249 (27.51)		0.015–1.030	0.056
Propoxur*	IN/AC	0.005	2 (0.22)	2 (0.22)	0.020–0.021	0.021
Pyridaben	IN/AC	0.15	21 (2.32)	–	0.015–0.150	0.034
Pyrimethanil	FU	0.8	5 (0.55)	–	0.028–0.450	0.127
Spinetoram*	IN	0.06	6 (0.66)	2 (0.22)	0.018–0.490	0.112
Spirodiclofen*	AC	0.1	3 (0.33)	–	0.016–0.076	0.038
Spiromesifen*	IN	0.3	33 (3.65)	–	0.015–0.110	0.028
Tebuconazole	FU	0.02	9 (0.99)	5 (0.55)	0.015–0.077	0.029
Thiamethoxam*	IN	0.01	7 (0.77)	7 (0.77)	0.012–0.046	0.020
Thiophanate-methyl*	FU	0.1	2 (0.22)	–	0.027–0.043	0.035
Valifenalate	FU	0.01	1 (0.11)	1 (0.11)	0.046	0.046

[a]AC: acaricide. [b]IN: insecticide. [c]FU: fungicide. [d]NE: nematicide. *Not approved in the EU.

Table S3. Chronic and acute hazard quotients (*HQc* and *HQa*) for pesticides detected in gherkin samples for adults and children.

			HQc (*UB*[a])		*HQa*	
**Pesticide**	***ADI*** **(mg kg^{-1} bw day^{-1})**	***ARfD*** **(mg kg^{-1} bw)**	**Adults**	**Children**	**Adults**	**Children**
Acequinocyl	0.023	0.08	7.0×10^{-5}	8.4×10^{-5}	0.0062	0.0149
Acetamiprid	0.005	0.005	2.9×10^{-4}	3.6×10^{-4}	0.4303	1.0292
Ametoctradin	10	–	1.5×10^{-7}	1.8×10^{-7}	0.0003	0.0007
Azoxystrobin	0.2	–	7.5×10^{-6}	9.0×10^{-6}	0.0112	0.0267
Bifenazate	0.01	0.1	1.6×10^{-4}	1.9×10^{-4}	0.0024	0.0057
Boscalid	0.04	–	4.0×10^{-5}	4.8×10^{-5}	0.0132	0.0316
Captan	0.25	0.9	6.4×10^{-6}	7.8×10^{-6}	0.0115	0.0275
Carbendazim	0.02	0.02	8.0×10^{-5}	9.7×10^{-5}	0.0003	0.0008
Chlorpyrifos	–	–	–	–	–	–
Clofentezine	0.017	–	9.3×10^{-5}	1.1×10^{-4}	0.0734	0.1756
Cyazofamid	0.17	–	9.4×10^{-6}	1.1×10^{-5}	0.0019	0.0046
Cyflumetofen	0.17	0.17	9.4×10^{-6}	1.1×10^{-5}	0.0047	0.0112
Cymoxanil	0.013	0.08	1.2×10^{-4}	1.5×10^{-4}	0.0061	0.0146
Cypermethrin	0.005	0.005	3.2×10^{-4}	3.9×10^{-4}	0.8197	1.9606
Deltamethrin	0.01	0.01	1.6×10^{-4}	1.9×10^{-4}	0.0434	0.1039
Dimethomorph	0.05	0.6	3.1×10^{-5}	3.8×10^{-5}	0.0019	0.0046
Etoxazole	0.04	–	4.0×10^{-5}	4.8×10^{-5}	0.0107	0.0257
Famoxadone	0.006	0.1	2.6×10^{-4}	3.2×10^{-4}	0.0042	0.0100
Flonicamid	0.025	0.025	5.2×10^{-5}	6.3×10^{-5}	0.0763	0.1824
Fluazinam	0.01	0.07	1.6×10^{-4}	1.9×10^{-4}	0.0029	0.0068
Fluopicolide	0.08	0.18	2.0×10^{-5}	2.4×10^{-5}	0.0036	0.0086
Fluopyram	0.012	0.5	1.3×10^{-4}	1.6×10^{-4}	0.0016	0.0038
Formetanate	0.004	0.005	4.0×10^{-4}	4.8×10^{-4}	0.0679	0.1624
Fosthiazate	0.004	0.005	4.0×10^{-4}	4.8×10^{-4}	0.0369	0.0884
Lambda-cyhalothrin	0.0025	0.005	6.4×10^{-4}	7.7×10^{-4}	0.0729	0.1743
Metalaxyl	0.08	0.5	2.0×10^{-5}	2.4×10^{-5}	0.0034	0.0082
Propamocarb	0.29	1	4.9×10^{-6}	5.9×10^{-6}	0.0051	0.0123
Propoxur	0.02	–	8.0×10^{-5}	9.7×10^{-5}	0.0052	0.0125
Pyridaben	0.01	0.05	1.6×10^{-4}	1.9×10^{-4}	0.0150	0.0358
Pyrimethanil	0.17	–	9.4×10^{-6}	1.1×10^{-5}	0.0132	0.0316
Spinetoram	0.025	0.1	6.4×10^{-5}	7.8×10^{-5}	0.0253	0.0605
Spirodiclofen	0.015	–	1.1×10^{-4}	1.3×10^{-4}	0.0245	0.0585
Spiromesifen	0.03	2	5.2×10^{-5}	6.3×10^{-5}	0.0003	0.0007
Tebuconazole	0.03	0.03	5.3×10^{-5}	6.4×10^{-5}	0.0128	0.0306
Thiamethoxam	0.026	0.5	6.1×10^{-5}	7.4×10^{-5}	0.0005	0.0011
Thiophanate-methyl	0.02	0.02	8.0×10^{-5}	9.7×10^{-5}	0.0107	0.0257
Valifenalate	0.07	–	2.3×10^{-5}	2.8×10^{-5}	0.0033	0.0078

[a]UB: Upper bound (results below the LOQ were replaced with the value of LOQ)

Table S4. MS/MS parameters (precursor ions, product ions, and collision energies) for target pesticides analysed by LC-MS/MS and GC-MS/MS.

**Pesticide**	**Analyzed by**	**Precursor ion (*m*/*z*)**	**Product ion (*m*/*z*)**	**CE (eV)**
2,4-D	LC-MS/MS	219.2	125.0	27
		219.2	161.0	15
2-phenylphenol	LC-MS/MS	170.1	170.1	22
		155.0	141.0	34
Acephate	LC-MS/MS	184.1	125.1	18
		184.1	143.0	8
Acequinocyl	GC-MS/MS	385.2	189.1	28
		385.2	343.2	4
Acetamiprid	LC-MS/MS	223.0	56.1	15
		223.0	126.0	20
Acibenzolar-s-methyl	LC-MS/MS	211.0	211.0	41
		136.1	91.1	29
Alachlor	GC-MS/MS	160.1	131.7	10
		160.1	188.1	8
Aldicarb	LC-MS/MS	208.2	89.1	19
		208.2	116.1	8
Aldicarb sulfone	LC-MS/MS	223.0	86.0	14
		223.0	148.0	10
Aldicarb sulfoxide	LC-MS/MS	207.0	89.0	14
		207.0	132.0	10
Ametoctradin	LC-MS/MS	276.3	149.1	39
		276.3	176.2	42
Amitraz	LC-MS/MS	294.4	163.2	14
		294.4	253.3	12
Atrazine	GC-MS/MS	226.3	184.2	19
		226.3	99.1	20
Azinphos-methyl	LC-MS/MS	317.9	159.9	6
		317.9	260.9	6
Azoxystrobin	LC-MS/MS	404.0	329.0	30
		404.0	372.0	15
Benfuracarb	LC-MS/MS	411.1	190.0	13
		411.1	195.0	23
Bensulfuron-methyl	LC-MS/MS	411.1	149.0	22
		411.1	182.0	20
Bentazone	LC-MS/MS	238.9	175.0	18
		238.9	196.9	19
Bifenazate	LC-MS/MS	301.0	198	11
		301.0	170	22
Bifenthrin	GC-MS/MS	181.1	160.1	25
		181.1	166.1	10
Boscalid	LC-MS/MS	342.9	139.9	20
		342.9	307.0	20
Bromoxynil	LC-MS/MS	275.8	78.7	17
		275.8	80.6	17
Bromuconazole	LC-MS/MS	376.0	70.1	25
		376.0	158.9	35
Bupirimate	LC-MS/MS	317.0	108.0	28
		317.0	166.0	28
Buprofezin	LC-MS/MS	306.1	57.4	20
		306.1	201.0	12
Cadusafos	LC-MS/MS	271.1	131.0	22

		271.1	159.0	16
Captan	GC-MS/MS	151.0	122.0	10
		151.0	80.0	10
Carbaryl	LC-MS/MS	202.0	117.0	28
		202.0	145.0	22
Carbendazim	LC-MS/MS	192.1	132.1	28
		192.1	160.1	18
Carbofuran	LC-MS/MS	222.1	123.0	16
		222.1	165.1	13
Carbosulfan	GC-MS/MS	381.3	160.2	9
		381.3	118.2	15
Carboxin	LC-MS/MS	236.0	87.0	22
		236.0	143.0	16
Chlorfluazuron	LC-MS/MS	539.8	158.0	20
		539.8	382.9	20
Chloridazon	LC-MS/MS	222.0	77.0	30
		222.0	92.0	30
Chlorpyrifos	LC-MS/MS	350.0	197.8	25
		350.0	97.1	32
Chlorpyrifos-methyl	GC-MS/MS	321.9	289.9	11
		321.9	125.0	17
Chlorsulfuron	LC-MS/MS	358.0	141.0	16
		358.0	167.0	18
Clethodim	LC-MS/MS	360.0	164.0	18
		360.0	268.1	12
Clodinafop	LC-MS/MS	350.0	91.0	32
		350.0	266.0	16
Clofentezine	LC-MS/MS	303.0	102.0	35
		303.0	138.0	22
Clothianidin	LC-MS/MS	250.0	132.0	18
		250.0	169.0	12
Cyazofamid	LC-MS/MS	325.2	108.1	14
		325.1	261.1	10
Cycloate	LC-MS/MS	216.0	134.0	6
		216.0	154.0	4
Cyflumetofen	LC-MS/MS	465.2	173	24
		465.2	249	16
Cymoxanil	LC-MS/MS	199.0	111.0	18
		199.0	128.0	8
Cypermethrin	LC-MS/MS	181.0	152.0	25
		181.0	127.0	35
Cyproconazole	LC-MS/MS	292.2	70.2	18
		292.2	125.1	24
Cyprodinil	LC-MS/MS	226.0	93.0	33
		226.0	108.0	25
Dazomet	LC-MS/MS	162.9	89.8	9
		162.9	119.9	14
Deltamethrin	GC-MS/MS	252.9	93.0	20
		252.9	171.9	8
Demeton-S-methyl	LC-MS/MS	231.1	61.2	30
		231.1	81.9	10
Demeton-S-methyl-sulfone	LC-MS/MS	263.0	121.0	17
		263.0	169.0	17
Demeton-S-methyl-sulfoxide	LC-MS/MS	247.0	109.0	25
		247.0	169.0	14

Desmedipham	LC-MS/MS	301.0	136.0	22
		301.0	182.0	10
Diafenthiuron	LC-MS/MS	385.4	278.3	33
		385.4	329.3	19
Diazinon	LC-MS/MS	305.0	97.0	35
		305.0	169.0	17
Dichlofluanid	GC-MS/MS	331.0	224.0	5
		331.0	123.0	25
Dichlorvos	LC-MS/MS	221.0	79.0	34
		221.0	109.0	22
Dicrotophos	LC-MS/MS	238.0	112.0	10
		238.0	193.0	10
Diethofencarb	LC-MS/MS	268.0	124.0	40
		268.0	226.0	10
Difenoconazole	LC-MS/MS	406.0	111.1	60
		406.0	251.1	25
Diflubenzuron	LC-MS/MS	309.0	155.9	11
		309.0	289.0	10
Dimethenamid	LC-MS/MS	276.0	168.0	26
		276.0	244.0	14
Dimethoate	LC-MS/MS	230.1	125.0	20
		230.1	199.0	10
Dimethomorph	LC-MS/MS	388.1	165.0	30
		388.1	300.9	20
Dinocap	LC-MS/MS	295.1	209.0	31
		295.1	193.4	28
Dinotefuran	LC-MS/MS	203.1	129.1	–15
		203.1	157.2	–12
Diuron	LC-MS/MS	233.0	46.3	14
		233.0	72.1	18
Dodine	LC-MS/MS	228.0	57.1	23
		228.0	60.1	23
Epoxiconazole	LC-MS/MS	330.0	101.0	50
		330.0	121.0	22
Ethiofencarb	LC-MS/MS	226.1	107.0	17
		226.1	164.0	8
Ethofumesate	LC-MS/MS	287.1	121.1	15
		287.1	259.1	10
Etofenprox	LC-MS/MS	394.3	106.9	43
		394.3	177.0	15
Etoxazole	LC-MS/MS	360.5	141.2	28
		360.5	177.3	20
Famoxadone	LC-MS/MS	392.2	238.0	20
		392.2	331.1	10
Fenamidone	LC-MS/MS	312.1	92.0	25
		312.1	236.1	14
Fenarimol	GC-MS/MS	330.8	267.9	20
		330.8	81.1	32
Fenazaquin	LC-MS/MS	307.2	57.2	25
		307.2	161.0	19
Fenbuconazole	LC-MS/MS	337.0	70.1	20
		337.0	125.0	36
Fenhexamid	LC-MS/MS	302.1	55.3	38
		302.1	97.2	22
Fenoxaprop-P-ethyl	LC-MS/MS	362.1	121.0	27

		362.1	287.9	18
Fenoxycarb	LC-MS/MS	302.1	88.0	20
		302.1	116.1	11
Fenpropathrin	LC-MS/MS	350.1	97.0	34
		350.1	125.0	14
Fenpyroximate	LC-MS/MS	422.2	138.1	32
		422.2	366.1	15
Fenthion	LC-MS/MS	279.1	169.1	16
		279.1	247.1	13
Flonicamid	LC-MS/MS	230	202.9	18
		230.1	148.1	28
Fluazinam	LC-MS/MS	463.0	398.0	20
		463.0	415.9	19
Fludioxonil	LC-MS/MS	247.0	180.0	28
		247.0	126.0	35
Flufenoxuron	LC-MS/MS	489.1	141.0	46
		489.1	158.0	22
Fluopicolide	LC-MS/MS	382.9	173	24
		382.9	144.9	55
Fluopyram	LC-MS/MS	397.1	172.9	33
		397.1	207.9	23
Flutriafol	LC-MS/MS	302.1	70.2	18
		302.1	123.1	29
Fonofos	GC-MS/MS	247.0	137.0	5
		247.0	109.0	15
Formetanate	LC-MS/MS	222.0	46.0	26
		222.0	165.0	15
Fosthiazate	LC-MS/MS	284.1	104	22
		284.1	227.9	12
Furathiocarb	LC-MS/MS	383.2	194.9	18
		383.2	252.0	12
Haloxyfop	LC-MS/MS	434.0	91.0	34
		434.0	316.0	20
Haloxyfop-P-methyl	LC-MS/MS	376.0	316.1	32
		376.0	288.0	25
Heptenophos	LC-MS/MS	251.0	125.0	14
		251.0	127.0	22
Hexaconazole	LC-MS/MS	314.0	70.1	28
		314.0	159.0	28
Hexaflumuron	LC-MS/MS	459.0	276.0	19
		459.0	439.0	12
Hexythiazox	LC-MS/MS	353.0	168.1	26
		353.0	228.1	14
Imazalil	LC-MS/MS	297.0	69.0	22
		297.0	159.0	22
Imidacloprid	LC-MS/MS	256.1	175.1	20
		256.1	209.1	15
Indoxacarb	LC-MS/MS	528.1	203.0	36
		528.1	150.0	16
Iodosulfuron	LC-MS/MS	508.1	141.1	25
		508.1	167.1	25
Ioxynil	LC-MS/MS	369.8	127.1	31
		369.8	215.1	30
Iprodione	GC-MS/MS	330.0	287.9	5
		330.0	245.0	12

Iprovalicarb	LC-MS/MS	320.8	119.1	–23
		320.8	203.1	–11
Kresoxim-methyl	LC-MS/MS	314.0	116.0	25
		314.0	206.0	40
Lambda-cyhalothrin	LC-MS/MS	467.0	141.0	45
		467.0	225.0	10
Lenacil	LC-MS/MS	235.2	136.1	32
		235.2	153.1	16
Linuron	LC-MS/MS	249.1	160.1	18
		249.1	181.1	16
Lufenuron	LC-MS/MS	509.2	175.0	40
		509.2	326.1	22
Malathion	LC-MS/MS	331.0	99.0	24
		331.0	127.0	12
Malaoxon	LC-MS/MS	315.0	98.9	24
		315.0	127.0	12
Mandipropamid	LC-MS/MS	412.0	328.0	–17
		412.0	356.1	–12
Mecarbam	LC-MS/MS	330.0	97.0	35
		330.0	227.1	8
Metalaxyl	LC-MS/MS	280.3	192.4	19
		280.3	220.4	14
Metamitron	GC-MS/MS	203.2	104.0	19
		203.2	42.0	29
Methacrifos	LC-MS/MS	241.1	125.0	20
		241.1	209.1	8
Methamidophos	LC-MS/MS	142.0	93.9	13
		142.0	124.9	13
Methiocarb	LC-MS/MS	226.0	169.0	22
		226.0	164.0	10
Methomyl	LC-MS/MS	162.9	87.9	9
		162.9	105.9	10
Metolachlor	LC-MS/MS	284.0	176.0	20
		284.0	252.0	8
Metribuzin	LC-MS/MS	215.0	89.0	20
		215.0	131.0	18
Mevinphos	LC-MS/MS	225.1	127.1	15
		225.1	193.1	8
Monocrotophos	LC-MS/MS	224.1	98.1	12
		224.1	127.1	16
Monolinuron	LC-MS/MS	215.0	99.0	34
		215.0	126.0	22
Myclobutanil	LC-MS/MS	289.1	70.2	18
		289.1	125.1	32
Nicosulfuron	LC-MS/MS	411.0	106.0	32
		411.0	182.0	22
Nuarimol	LC-MS/MS	315.0	81.1	28
		315.0	252.0	22
Omethoate	LC-MS/MS	214.1	125.1	22
		214.1	183.1	11
Oxadixyl	LC-MS/MS	279.0	132.0	34
		279.0	219.0	10
Oxamyl	LC-MS/MS	237.0	72.0	10
		237.0	90.0	10
Oxyfluorfen	GC-MS/MS	362.0	237.0	20

		362.0	316.0	5
Paraoxon-ethyl	LC-MS/MS	276.4	174.3	25
		276.4	220.3	14
Penconazole	LC-MS/MS	284.0	70.1	16
		284.0	159.0	34
Pendimethalin	LC-MS/MS	282.2	194.1	17
		282.2	212.2	10
Phenmedipham	LC-MS/MS	301.0	136.0	22
		301.0	168.0	10
Phenthoate	LC-MS/MS	321.0	135.0	20
		321.0	163.0	12
Phorate	GC-MS/MS	261.0	74.9	8
		261.0	170.9	12
Phosalone	LC-MS/MS	367.9	110.9	42
		367.9	181.9	14
Phosphamidon	LC-MS/MS	300.1	127.1	25
		300.1	174.1	14
Phosmet	LC-MS/MS	318.0	77.0	46
		318.0	160.0	22
Pirimicarb	LC-MS/MS	239.1	72.0	18
		239.1	182.1	15
Pirimiphos-ethyl	LC-MS/MS	334.2	198.1	19
		334.2	182.1	19
Pirimiphos-methyl	LC-MS/MS	306.0	108.0	31
		306.0	164.0	19
Prochloraz	LC-MS/MS	376.2	70.1	34
		376.2	308.1	11
Profenofos	LC-MS/MS	373.0	97.0	35
		373.0	303.0	15
Profoxydim	LC-MS/MS	46.5	180.3	26
		46.5	280.3	24
Promecarb	GC-MS/MS	208.1	109.0	15
		208.1	151.0	9
Prometryn	LC-MS/MS	242.0	158.0	25
		242.0	200.1	17
Propamocarb	LC-MS/MS	189.1	102.0	17
		189.1	144.0	22
Propaquizafop	LC-MS/MS	444.2	100.0	20
		444.2	163.1	60
Propargite	LC-MS/MS	368.4	57.1	20
		368.4	231.3	8
Propazine	LC-MS/MS	230.2	146.1	24
		230.2	188.1	18
Propiconazole	LC-MS/MS	342.0	69.0	22
		342.0	159.0	34
Propoxur	LC-MS/MS	210.0	111.0	16
		210.0	168.0	10
Propyzamide	LC-MS/MS	256.1	173.0	23
		256.1	190.0	16
Prosulfuron	LC-MS/MS	418.1	139.1	22
		418.1	252.1	14
Prothiofos	LC-MS/MS	345.1	133.0	56
		345.1	241.0	18
Pymetrozine	LC-MS/MS	218.0	79.0	30
		218.0	105.0	20

Pyrazophos	LC-MS/MS	374.0	194.0	33
		374.0	222.0	17
Pyridaben	LC-MS/MS	365.1	147.1	24
		365.1	309.1	12
Pyridaphenthion	LC-MS/MS	341.0	92.0	34
		341.0	189.0	22
Pyridate	LC-MS/MS	379.0	207.0	18
		379.0	351.1	10
Pyrimethanil	LC-MS/MS	199.8	107.1	28
		199.8	82.2	30
Pyriproxyfen	LC-MS/MS	322.1	96.0	14
		322.1	227.1	14
Rimsulfuron	LC-MS/MS	431.9	182.1	22
		431.9	325.1	14
Sethoxydim	LC-MS/MS	328.0	178.0	22
		328.0	282.0	10
Simazine	GC-MS/MS	202.1	124.0	13
		202.1	104.0	13
Spinosyn A	LC-MS/MS	732.6	98.1	59
		732.6	142.0	31
Spinosyn D	LC-MS/MS	746.5	98.1	53
		746.5	142.0	31
Spinetoram	LC-MS/MS	748.4	142.1	–37
		748.4	98.2	–55
Spirodiclofen	LC-MS/MS	411.2	71.2	22
		413.1	315.1	14
Spiromesifen	LC-MS/MS	371.1	273.2	12
		371.1	255.2	26
Spiroxamine	LC-MS/MS	298.0	100.0	32
		298.0	144.0	20
Tau-fluvalinate	GC-MS/MS	250.1	155.0	20
		250.1	200.0	20
Tebuconazole	LC-MS/MS	308.0	70.1	22
		308.0	125.0	40
Tebufenozide	LC-MS/MS	353.1	133.0	20
		353.1	297.1	8
Tebufenpyrad	LC-MS/MS	334.0	117.0	34
		334.0	145.0	28
Teflubenzuron	LC-MS/MS	379.0	339	11
		379.0	358.9	6
Tepraloxydim	LC-MS/MS	342.1	166.1	20
		342.1	250.1	12
Terbufos	GC-MS/MS	288.9	103.0	2
		288.9	57.1	18
Terbuthylazine	LC-MS/MS	230.0	96.0	28
		230.0	174.0	16
Terbuthylazine-desethyl	LC-MS/MS	202.1	79.1	26
		202.1	146.1	16
Terbutryn	LC-MS/MS	242.0	68.0	45
		242.0	186.0	13
Tetraconazole	LC-MS/MS	372.0	70.1	20
		372.0	159.0	30
Thiabendazole	LC-MS/MS	202.0	131.0	30
		202.0	175.0	25
Thiacloprid	LC-MS/MS	253.0	90.1	40

		253.0	126.0	20
Thiamethoxam	LC-MS/MS	292.0	132.0	22
		292.0	211.2	12
Thifensulfuron-methyl	LC-MS/MS	388.0	56.0	40
		388.0	167.0	15
Thiobencarb	LC-MS/MS	258.2	89.1	48
		258.2	125.1	18
Thiodicarb	LC-MS/MS	355.0	87.9	16
		355.0	107.9	16
Thiophanate-methyl	LC-MS/MS	343.0	93.0	46
		343.0	151.0	22
Tolylfluanid	LC-MS/MS	364.0	137.0	25
		364.0	238.0	1
Tralkoxydim	LC-MS/MS	330.2	138.1	19
		330.2	284.3	13
Triadimefon	LC-MS/MS	294.1	69.3	20
		294.1	197.2	15
Triadimenol	LC-MS/MS	296.1	70.2	10
		296.1	99.1	15
Tri-allate	LC-MS/MS	304.0	86.0	18
		304.0	142.9	28
Triasulfuron	LC-MS/MS	402.0	141.0	20
		402.0	167.1	17
Trichlorfon	LC-MS/MS	257.0	79.0	30
		257.0	109.0	18
Trifloxystrobin	LC-MS/MS	409.0	145.0	40
		409.0	186.0	16
Triflumizole	LC-MS/MS	346.0	73.1	10
		346.0	277.9	10
Triflumuron	LC-MS/MS	356.9	154	13
		356.8	84.7	45
Triticonazole	LC-MS/MS	318.1	70.1	16
		318.1	124.9	35
Valifenalate	LC-MS/MS	399.2	154.9	40
		401.2	116.0	29
Vamidothion	LC-MS/MS	288.0	118.1	28
		288.0	146.	10
Vinclozolin	GC-MS/MS	285.0	212.0	12
		285.0	178.0	14

*CE: Collision energy (eV)

Table S5. Definition and individual scores of indices for the pesticide residual risk ranking.

**Categories**	**Definition**	**Score**	**Definition**	**Score**	**Definition**	**Score**	**Definition**	**Score**
A: Toxicity (LD_{50})	Low toxicity	2	Moderate toxicity	3	Highly toxicity	4	Rank poison	5
B: Toxic effect (ADI)	＞0.01	0	0.0001–0.01	1	0.000001–0.0001	2	＜0.000001	3
C: Proportion of diet (%)	＜2.5	0	2.5–20	1	20–50	2	50–100	3
D: Frequency of pesticide (%)	＜2.5	0	2.5–20	1	20–50	2	50–100	3
E: High-exposure crowd	nothing	0	few	1	mainly	2	indeterminacy	3
F: Residual level	ND	1	＜1MRL	2	1–10MRL	3	≥10MRL	4

Table S6. Assigned scores for indices A-F used in the pesticide residue risk scoring system.

Pesticide	Toxicity (LD_{50}) A	Toxic effect (ADI) B	Proportion of diet C	Frequency of pesticide D	High-exposure crowd E	Residual level (mg/kg)* F
Acequinocyl	2	1	0	1	3	1.00
Acetamiprid	3	1	0	1	3	1.13
Ametoctradin	2	0	0	1	3	1.09
Azoxystrobin	2	0	0	1	3	1.11
Bifenazate	2	1	0	1	3	1.01
Boscalid	2	0	0	1	3	1.01
Carbendazim	2	0	0	0	3	1.01
Chlorpyrifos	3	–	0	0	3	–
Captan	2	0	0	1	3	1.00
Clofentezine	2	0	0	1	3	1.02
Cyazofamid	2	0	0	1	3	1.00
Cyflumetofen	2	0	0	1	3	1.01
Cymoxanil	3	0	0	1	3	1.00
Cypermethrin	2	1	0	1	3	1.01
Deltamethrin	3	0	0	1	3	1.00
Dimethomorph	2	0	0	0	3	1.03
Etoxazole	2	1	0	1	3	1.02
Famoxadone	2	0	0	0	3	1.04
Flonicamid	3	0	0	1	3	1.36
Fluazinam	2	1	0	1	3	1.00
Fluopicolide	2	0	0	1	3	1.03
Fluopyram	2	0	0	1	3	1.04
Formetanate	4	1	0	1	3	1.00
Fosthiazate	2	1	0	1	3	1.01
Lambda-cyhalothrin	3	1	0	1	3	1.01
Metalaxyl	3	0	0	1	3	1.06
Propamocarb	2	0	0	1	3	1.28
Propoxur	3	0	0	0	3	1.00
Pyridaben	3	1	0	1	3	1.02
Pyrimethanil	2	0	0	1	3	1.01
Spinetoram	2	0	0	0	3	1.01
Spirodiclofen	2	0	0	0	3	1.00
Spiromesifen	2	0	0	0	3	1.04
Tebuconazole	3	0	0	1	3	1.02
Thiamethoxam	3	0	0	0	3	1.02
Thiophanate-methyl	2	0	0	0	3	1.00
Valifenalate	2	0	0	1	3	1.00

*Pesticides with the content exceeding the detection limit (LOQ) participated in the calculation.
